# Orientation of reduced graphene oxide in composite coatings†

**DOI:** 10.1039/d3na01057k

**Published:** 2024-03-11

**Authors:** Knut Thorshaug, Terje Didriksen, Ingvild Thue Jensen, Patricia Almeida Carvalho, Juan Yang, Mathieu Grandcolas, Alain Ferber, Andy M. Booth, Özlem Ağaç, Hüseyin Alagöz, Nursev Erdoğan, Anıl Kuban, Branson D. Belle

**Affiliations:** a SINTEF Industri Forskningsveien 1 NO-0373 Oslo Norway knut.thorshaug@sintef.no; b SINTEF Digital Forskningsveien 1 NO-0373 Oslo Norway; c SINTEF Ocean Brattørkaia 17C NO-7010 Trondheim Norway; d Nanografi Nanotechnology AS ODTÜ Teknokent No: 13/1-1 06531 Çankaya Ankara Turkey; e Turkish Aerospace, Functional Coatings & Transparencies Technology Centre Ankara Turkey

## Abstract

Composite coatings containing reduced graphene oxide (rGO) and 3-(aminopropyl)triethoxysilane functionalised rGO (APTES-rGO) were prepared by sol–gel technology and deposited on Al 2024 T-3. Covalent functionalisation of GO by APTES occurred by formation of amide bonds, accompanied by GO reduction. The thin sheets were retained. The hydrophilicity of the coating increased when APTES-rGO was added. The opposite was observed when GO was added. A key finding is that the rGO flakes agglomerate and lie in a random orientation in the coating, whereas the APTES-rGO flakes are more evenly distributed in the matrix and appear to lie along the plane of the substrate.

## Abstract 2

In composite coatings containing 3-(aminopropyl)triethoxysilane functionalised rGO (APTES-rGO), the flakes are distributed in the matrix and appear to lie along the plane of the substrate.

## Graphical abstract

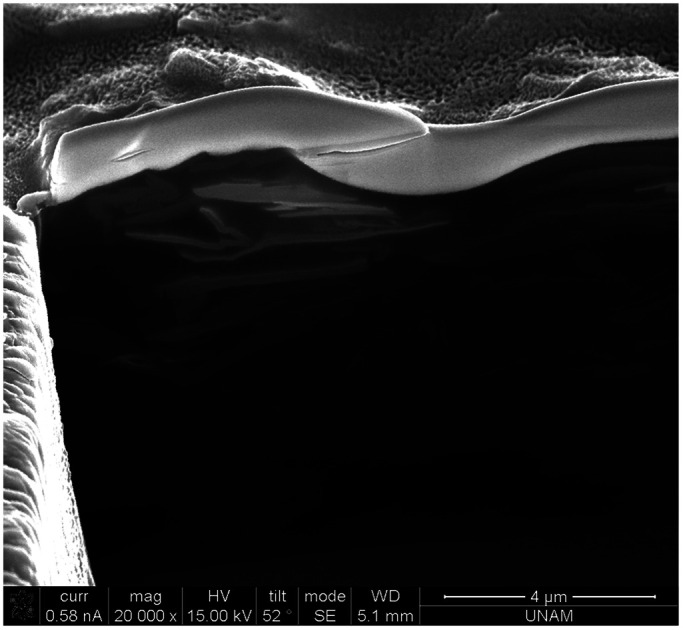

## Introduction

Surface modification is important in a myriad of settings, and functional coatings have been developed for numerous purposes, *e.g.*, anti-bacterial,^1^ anti-icing,^2^ self-cleaning,^3^ anti-fouling,^4^ and anti-corrosion.^5^ Graphene oxide (GO) and its derivatives have been utilised as coating components due to GO's impermeability.^6^ While impermeability has been extensively explored,^7^ it has also been noted that GO dispersibility issues may arise.^8^

GO is a 2D material, and thus its orientation in a coating should be considered. For example, recent simulations of graphene flake orientation in composite coatings showed that a low angle between the sheets and substrate is beneficial because it delays corrosion onset.^9^ Furthermore, alternately stacked reduced GO (rGO) sheets suppress gas molecule permeation,^10^ while the horizontal alignment of ultra large GO sheets influences the moisture barrier properties in polyurethane composites,^11^ and fuel permeability is related to the orientation of GO in a Nafion/GO composite membrane.^12^ Controlled GO alignment in nanocomposite films was achieved by emulsion polymerisation protocols, and alignment was demonstrated to influence material properties, *e.g.*, electrical conductivity.^13–15^ GO flake alignment has also been achieved by magnetically induced orientation^16^ and solid drawn composite films.^17^

Various coatings have been reported without mentioning the GO flake orientation. For instance, rGO has been successfully employed in protective coatings reported to be highly impermeable to gasses, liquids, and aggressive chemicals, including HF.^18^ Nanocomposites containing GO and rGO have been developed,^19–22^ and a comparison of nanocomposites prepared using chemically functionalised GO and nanocomposites produced as physical blends, showed the former to be superior.^8^ Nanocomposites incorporating rGO platelets functionalised by 3-(aminopropyl)triethoxysilane (APTES) yielded a corrosion protection layer on Al T-2024.^23^ Similarly, an anti-corrosion coating for the AA-2024 Al alloy was prepared by grafting rGO with 3-(glycidyloxypropyl)-trimethoxysilane (GPTMS).^24^ APTES functionalised GO was used as additive in the preparation of nanocomposites with improved thermal conductivity,^25^ and flux-enhanced mixed matrix membranes.^26^ For GO containing composite coatings, the dispersion and stability in the organic matrix have been demonstrated to be significantly influenced by covalent GO functionalisation.^27^

In the current study, we present and discuss coatings that contain rGO, and APTES functionalised rGO (APTES-rGO), respectively, and we provide evidence for APTES-rGO flake alignment.

## Experimental

### Materials

A 0.5 wt% aqueous dispersion of graphene oxide (GO) was used as received from Nanografi. APTES, GPTMS, 2-propanol, dicyclohexylcarbodiimide, phenyltrimethoxysilane (Ph(OMe)_3_Si), HNO_3_, and Zr(acetylacetonate)_4_ (Zr(acac)_4_) were used as received from Sigma-Aldrich. Al 2024 T-3 provided by Turkish Aerospace (TA) was used after cleaning with methylethylketone (MEK), followed by immersion in Bonderite, basic, and acid baths, respectively. Di-water rinse and water-break tests were performed between each of the latter three baths.

### Functionalisation

APTES-rGO was prepared by modification of the procedure described by Hu *et al.*^28^ A mixture of graphene oxide (0.50 g), dicyclohexylcarbodiimide (0.50 g), and APTES (7.5 g) in THF (250 ml), was sonicated for 15 min and then heated to reflux. After 48 h, the mixture was cooled to room temperature, concentrated to *ca.* 100 ml on a rotary vapor, and diluted with methanol (900 ml). The mixture was then stirred and sonicated before filtration through a 0.45 μm PVDF membrane (Millipore filtration). The product was washed with hot THF and hot methanol, and then sonicated with 100 ml THF for 15 min. The methanol treatment, and subsequent filtering and washing was repeated once more. The product was first air-dried on the filter and, then under vacuum (<0.1 mbar) overnight. Yield: 0.85 g.

### Sol–gel

A solution of GPTMS (4.83 g), APTES (4.49 g), Ph(OMe)_3_Si (10.16 g), 2-propanol (20.04 g), and Zr(acac)_4_ (0.48 g) was stirred at room temperature overnight. HNO_3_ (0.01 N, 2.60 g), and 2-propanol (6 g) were added, and the solution turned weakly yellow while stirring.

### Spray coating

Sol–gel and an aqueous dispersion of GO were mixed in the desired ratio, stirred, and poured into the liquid reservoir on a spray gun. The liquid was manually sprayed onto the Al 2024 T-3 substrates. Finally, the coatings were cured at 80 °C for two hours.

### Fourier transform infrared (FTIR) spectroscopy

Fourier Transform Infrared (FTIR) spectroscopy was carried out on a Bruker Vertex 70 equipped with both a DTGS- and an MCT-detector. Potassium bromide pellets were prepared for the transmission mode studies, while a set-up from Bruker was used for reflectance mode analysis. In both modes we used 16 scans, and 4 cm^−1^ resolution.

### Raman spectroscopy

Raman spectroscopy was carried out on a BWS465-785S Tek i-Raman Plus instrument fitted with a 785 nm probe.

### Atomic force microscopy (AFM)

Atomic Force Microscopy (AFM) was carried out on a Bruker Multimode 8 using peak force tapping mode. Scan Asyst silicon nitride tips with a nominal spring constant of 0.4 N m^−1^ were used.

### Scanning electron microscopy (SEM)

Scanning Electron Microscopy (SEM) was carried out on a FEI Nova NanoSEM 650.

### X-ray photoelectron spectroscopy (XPS)

X-ray Photoelectron Spectroscopy (XPS) was carried out using a Kratos AXIS UltraDLD instrument, with monochromatic Al Kα radiation (*hν* = 1486.6 eV) operated at 10 A and 15 kV. Survey spectra were collected with step size 1 eV and pass energy (PE) 160 eV. Core peaks were measured with step size 0.1 eV and PE 40 eV. Charge correction was applied using a low energy electron flood gun. The spectra were fitted using CasaXPS^29^ after Shirley background subtraction.^30^ The C 1s component of adventitious carbon was set to 284.8 eV to calibrate the energy scale of the charge corrected spectra.

### Focused ion beam scanning electron microscopy (FIB SEM)

Focused Ion Beam Scanning Electron Microscopy (FIB SEM) was carried out on a Helios G4 UX dual-FIB. A thin layer of Pt/Pd was sputter coated onto the sample prior to FIB sample preparation. An ion-beam acceleration voltage of 30 kV was used to prepare the cross-section.

### Water contact angles

Water contact angles were measured using an OSSILA goniometer. A 10 μL deionised water drop was used for each measurement and the average contact angle was calculated from several measurements for each sample.

## Results and discussion

### GO and APTES-rGO

**Scheme 1:**
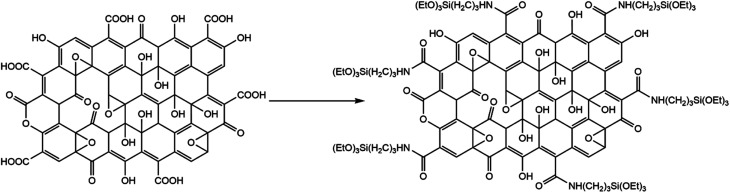
Preparation of APTES-rGO from GO, drawing based on information in Aliyev *et al.*^33^

**Fig. 1:**
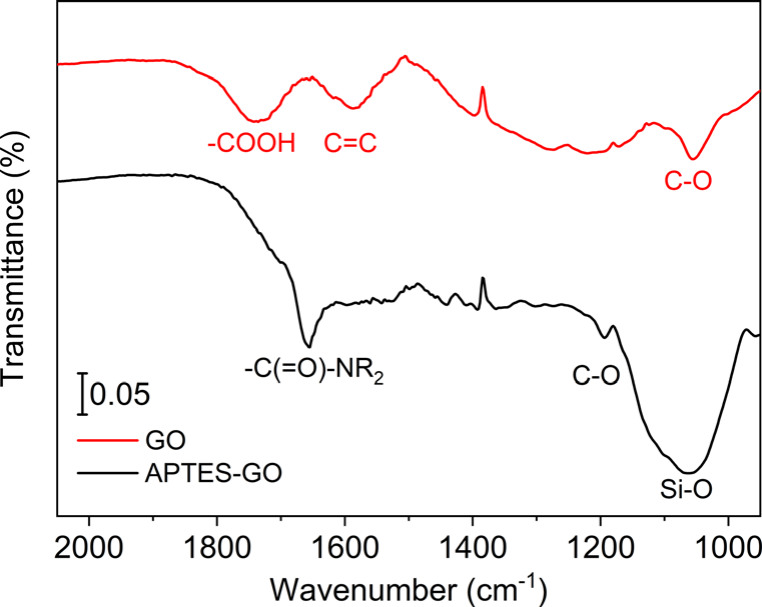
FTIR spectra of GO (upper, red) and APTES-rGO (lower, black).

GO (Scheme 1) is known to contain various functional groups,^31–33^ and the FTIR spectrum collected from GO used herein (Fig. 1) compares well to the spectrum reported earlier for multilayer GO.^34^ The broad band centred at 1738 cm^−1^ is assigned to carboxyl groups located on the edges of the sheets,^35^ while bands at 1221 cm^−1^ and 1052 cm^−1^ are assigned to epoxy CO, and alkoxy CO, respectively.^36^

APTES functionalisation of GO (Scheme 1) was achieved by modification of Hu *et al.*'s method.^28^ Hu *et al.*'s^28^ protocol reproducibly delivered a material that could not be dispersed. We tweaked the procedure by keeping the modified GO dispersed throughout the work-up, and thereby obtained the final product as a stable dispersion.

We expected GO functionalisation by APTES to occur by a reaction between –COOH and –NH_2_ in GO and APTES, respectively, yielding an amide functionality. The FTIR spectrum shows a newly formed band centred at 1657 cm^−1^ in the reaction product (Fig. 1), supporting the view that functionalisation indeed occurred by formation of an amide fragment. XPS further supports this view, as described further below. Furthermore, the FTIR bands assigned to functional groups in GO were lower in intensity after functionalisation, most likely due to GO reduction. The material after functionalisation is thus best described as APTES-rGO.

The observed consumption of the band at 1738 cm^−1^ assigned to –COOH groups in GO supports the view that –COOH is the site where functionalisation occurs, although the weak shoulder at *ca.* 1700 cm^−1^ suggests that some unreacted carbonyl remains after functionalisation. Some reaction may also have occurred on the epoxide groups of the GO, as reported by Pu *et al.*^25^ for a reaction done under similar conditions. The strong band at *ca.* 1050 cm^−1^ confirms the presence of Si–O.

**Fig. 2:**
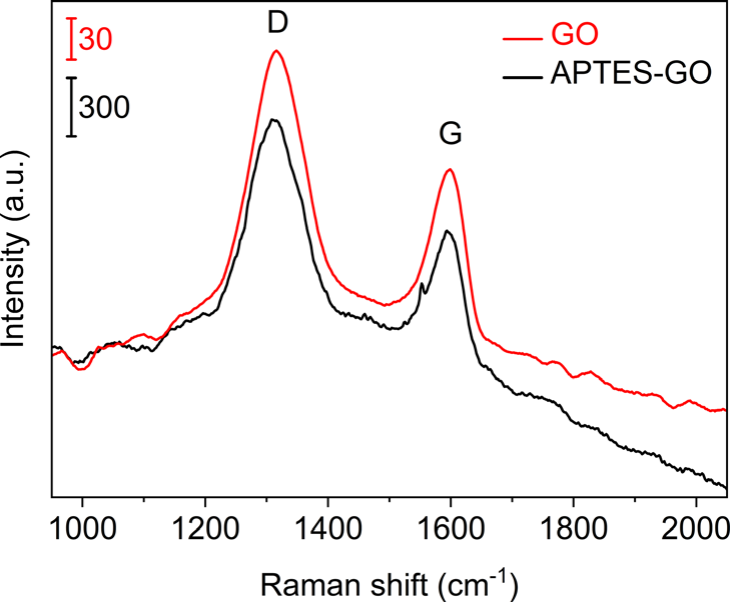
Raman spectra of GO (upper, red) and APTES-rGO (lower, black). Note the different vertical bars.

Raman spectra (Fig. 2) collected from GO and APTES-rGO both showed characteristic D and G bands at 1315 cm^−1^ and 1592 cm^−1^, in reasonable agreement with the values reported by Melucci *et al.*^37^ The D band position is known to depend on the laser excitation, and 1315 cm^−1^ compares well to the value 1308 cm^−1^ reported by McCreary *et al.*^38^ for the same laser excitation as used herein.

**Fig. 3:**
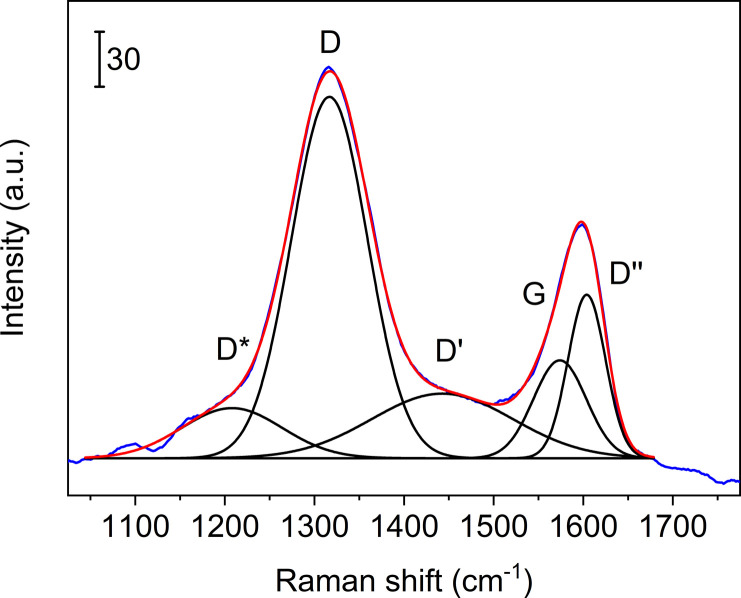
Curve-fits of five bands to the Raman spectrum collected from rGO in the coating. Blue line: experimental data, red line: sum of fits, black lines: curve fits.

More details are available from the Raman spectra, as there are five bands in the range 1000–1750 cm^−1^ that are of particular interest.^39–41^Fig. 3 illustrates the fits obtained for rGO, and in Table 1 we summarise the results from curve-fitting to each Raman spectrum. The fitted spectra are shown in Fig. S6–S9 in the ESI.† By comparing the G-bands for GO and APTES-rGO (Table 1), we see that APTES-functionalisation induced a 12 cm^−1^ blue shift compared to GO. The blue shifted G band may be attributed to the amide groups formed by APTES functionalisation, and the G band shift reported herein compares well to the 8 cm^−1^ G band blue shift observed for GO and octadecylamine-GO.^42^ The *I*_D_/*I*_G_ ratio calculated from the band intensities was approximately 1.55 for both samples.

**Table 1:** Curve-fitted Raman shifts. All numbers are in cm^−1^

	GO	APTES-rGO	In coating	
			rGO	APTES-rGO
D*	1208	1276	1267	1176
D	1317	1313	1317	1314
D′′	1443	1471	1465	1443
G	1574	1562	1574	1555
D′	1604	1601	1607	1599

**Fig. 4:**
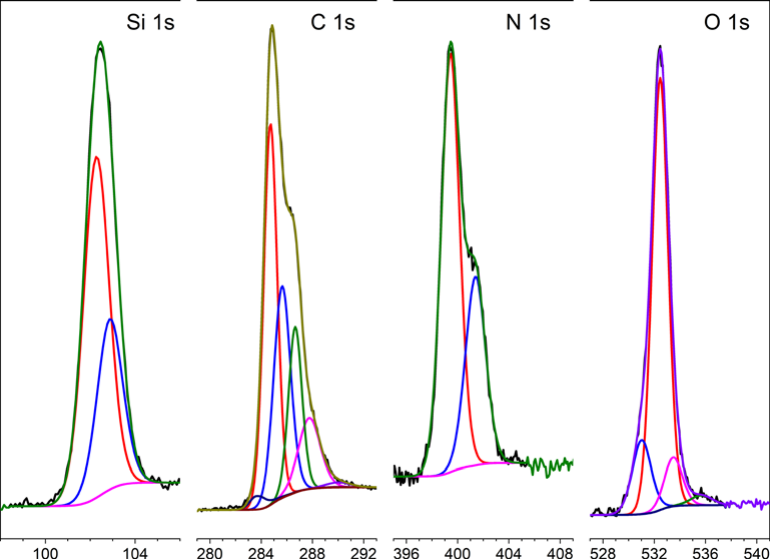
XPS-spectra collected from APTES-rGO.

The XPS-spectra of APTES-rGO are shown in Fig. 4, whereas those of GO are provided in the ESI.† For APTES-rGO, the C 1s signal at 288.4 eV and the N 1s signal at 401.5 eV both compare well to the values 288.5 eV and 401.4 eV, reported for an N–C=O fragment in highly exfoliated GO functionalised by l-arginine, aniline, and β-alanine.^43^ Furthermore, the two N 1s peaks at 399.4 eV and 401.4 eV, respectively, and the Si 1s peak at 103.4 eV, which can be deconvoluted into two peaks at 102.3 eV and 102.9 eV, all correspond well to the data reported by Serordre *et al.*^44^ Finally, the O 1s peak centred at 532.5 eV can be deconvoluted into three components centred at 531.0 eV, 532.5 eV, and 533.5 eV, respectively. Thus, a complete set of XPS peaks for APTES-rGO is now available. The XPS data and the FTIR spectra both confirm covalent APTES-functionalised rGO.

**Fig. 5:**
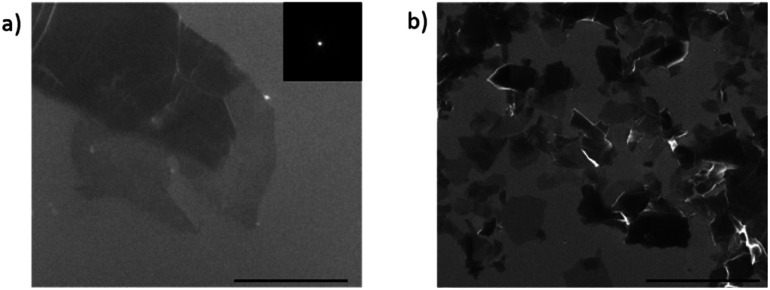
SEM micrograph of (a) GO and (b) APTES-rGO. The indicated scale bar is 2 μm.

Fig. 5 shows SEM micrographs of GO and APTES-rGO. The GO samples exhibit large flakes with thin regions as shown by the areas of light contrast. By contrast, the APTES-rGO samples have a greater distribution of crystallites with varying thicknesses due to added exfoliation during the APTES functionalisation process.

GO and APTES-rGO sheets were observed by AFM and they are shown in (Fig. 8). As seen, the GO sample consists of agglomerations of flakes. Monolayer edges can be found. The line trace shows a substrate to flake step height of around 1.3 nm. Although this step height is larger than the interlayer spacing for graphene, it is customary to have an additional step height due to water and functional groups trapped between the GO flake and the substrate. For APTES-rGO, thin sheets are also easily observable however these sheets standoff of the surface giving larger thicknesses due to the APTES functionalisation. Again, here an assumption of a standoff height of about 1 nm results in a layer thickness between 1 and 4 layers.

The concurrent reduction of GO during APTES functionalisation can be seen from the TUNA current maps (Fig. S20 and S21, ESI†). The GO flakes are found to be insulating and non-conducting, whereas for some APTES-rGO flakes there are areas with increased conductivity. Reduction of GO to rGO during APTES functionalisation compares well to Pu *et al.*,^25^ although we report functionalisation to occur mainly on the carboxylic groups on the GO edge although we cannot rule out reactions on the epoxides on the GO plane. Pu *et al.*,^25^ on the other hand, claim the reaction site to be epoxides on the GO plane.

### Coatings containing rGO and APTES-rGO

**Fig. 6:**
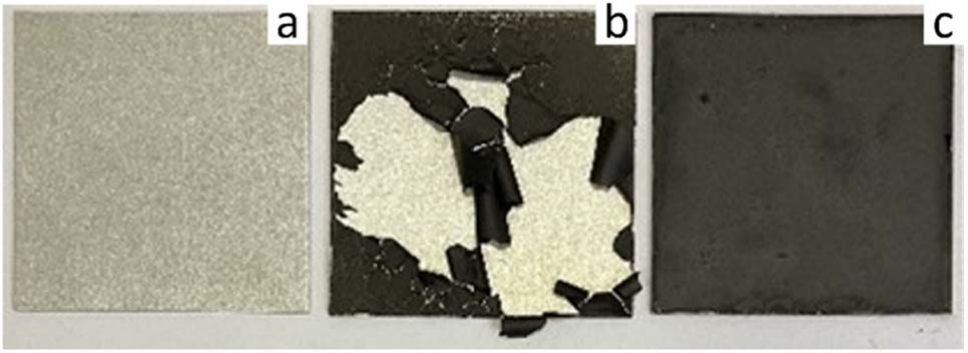
(a) Clean Al 2024 T-3, (b) GO on Al 2024 T-3, (c) 5 wt% rGO in coating on Al 2024 T-3 (right). Sample size 5 × 5 cm.

When aqueous dispersions of GO and APTES-rGO were coated onto clean Al 2024 T-3 substrates and dried, the adhesion to Al was poor. For both samples, the coating peeled off during curing as illustrated for GO in Fig. 6.

**Fig. 7:**
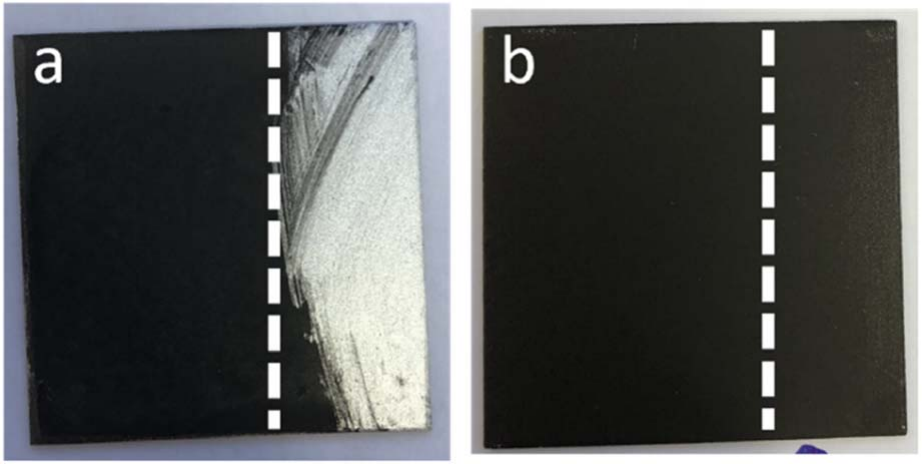
Coatings on Al 2024 T-3. (a) APTES-rGO on sol–gel and (b) APTES-rGO in sol–gel. Vertical dotted lines are guides to the eye only. Wipe with moist cloth on the right side of the dotted line. Sample size = 5 × 5 cm.

The situation improved significantly when GO and APTES-rGO were incorporated into coatings prepared by sol–gel methodology. By mixing GO or APTES-rGO with a solution made from GPTMS, APTES, Ph(OMe)_3_Si, 2-propanol, Zr(acac)_4_, and HNO_3_, followed by spray coating onto an Al-coupon and heat treatment at 80 °C for two hours, the coatings were found to adhere well to the surface. The sol–gel coating is clear in the absence of additives, thus, the dark colouring stems from the presence of rGO and APTES-rGO in the coating (Fig. 7). The thickness was measured to be 9 μm and 6 μm for the coating containing rGO and APTES-rGO, respectively (ESI†).

**Fig. 8:**
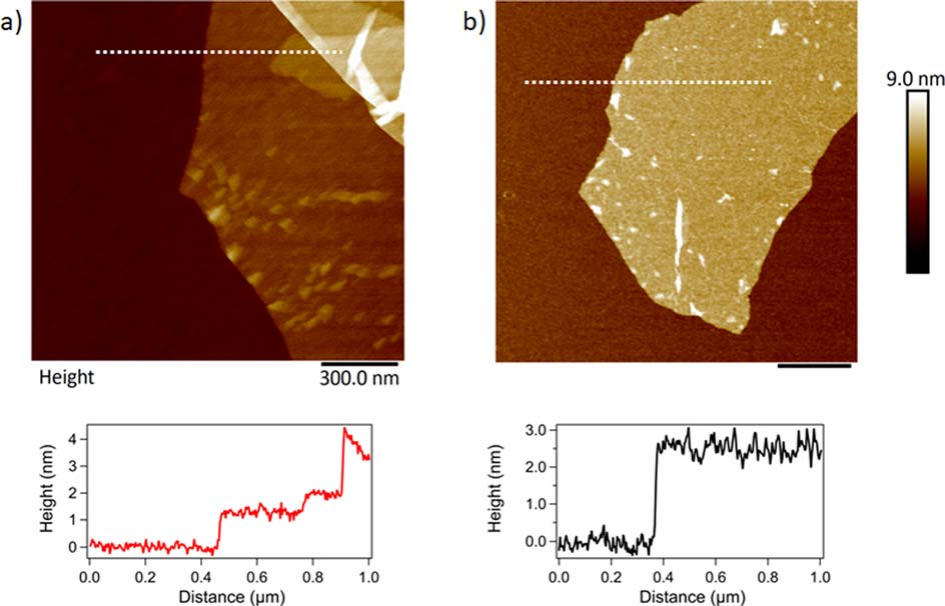
. AFM topography images of (a) GO and (b) APTES-rGO. The scale bar is 300 nm in both images. Line traces show the step height along the dotted line in (a) and (b).

In principle, at least two different coatings can be prepared from GO and the sol–gel precursor solution. One approach is to mix GO into the solution before it is sprayed onto the substrate, yielding a hybrid coating with a uniform distribution of GO. The second approach is to first spray the solution onto the substrate, and then spray GO on top of the first coating layer. This would yield a two-layer coating with GO on the outer surface. In this study, only the first approach resulted in a coating that adhered well to the Al surface. For coatings prepared by the second approach, the outer GO layer could easily be wiped off with a moist cloth leaving behind an essentially non-GO containing coating (Fig. 7). We therefore opted to focus on the hybrid coatings prepared by the first approach, *i.e.*, pre-mixing of GO or APTES-rGO into the solution before spray coating. Images of clean Al 2024 T3 together with the surface coated with sol–gel with no additives, rGO added, and APTES-rGO added are shown in the ESI (Fig. S20†).

**Fig. 9:**
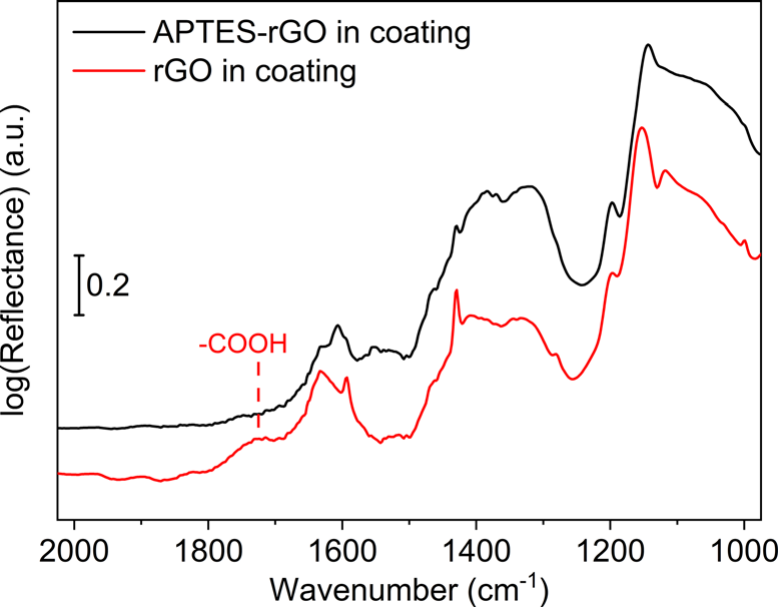
FTIR spectra. rGO in coating (red, lower), and APTES-rGO in coating (upper, black).

FTIR spectra of the coatings are shown in Fig. 9. Although the sol–gel components dominate the spectra, we see that the band observed at 1738 cm^−1^ in the GO (Fig. 1) is consumed when GO is incorporated into the coating, thus, GO reduction takes place. A broad band is detected at *ca.* 1722 cm^−1^ which we assign to remaining carboxylic groups in the sample.

**Fig. 10:**
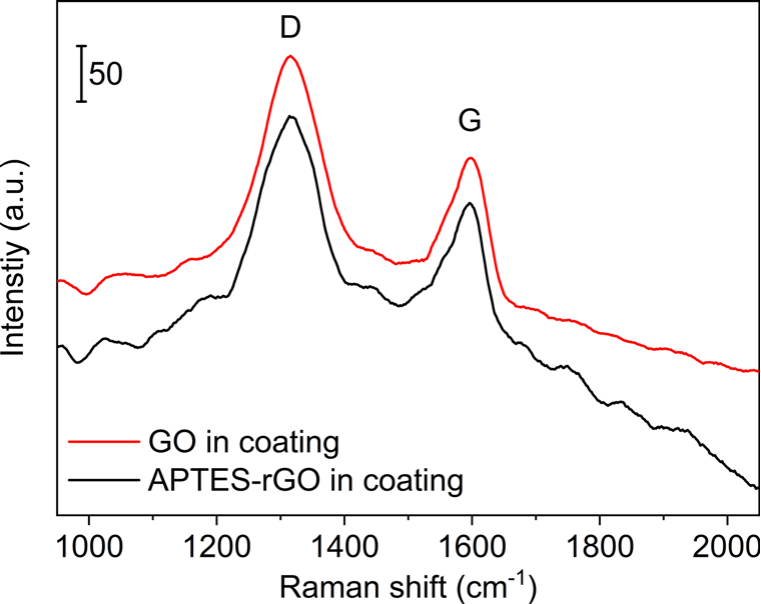
Raman spectra of coatings containing rGO and APTES-rGO.

For GO the position of the D and G-bands were found to be centred at 1317 cm^−1^ and 1574 cm^−1^, respectively, both before and after GO was incorporated into the coating (Fig. 10). For GO, the *I*_D_/*I*_G_-ratio was calculated to be 1.64 in the coating, compared to 1.55 in the starting GO material. The increase is in line with a reduction taking place when GO is incorporated in the coating and thus rGO is indeed the correct description. For APTES-rGO, the *I*_D_/*I*_G_-ratio was found to be 1.51 in the coating which is comparable to 1.55 found in the starting APTES-rGO.

Fig. 3 illustrates the fits obtained for rGO, and Table 1 is a summary of the curve-fitting to each spectrum shown in Fig. 2 and 10. As seen from Table 1, the D, G, and D′ peak positions do not shift to any significant degree when the graphene oxide materials are embedded in the coating, except for the 7 cm^−1^ shift in the APTES-rGO G band. The D* and D′′ band positions, on the other hand, vary significantly. Incorporation of rGO and APTES-rGO in the coating shift the D* peak positions +59 cm^−1^ and −100 cm^−1^, whereas the D′′ peak positions shift +22 cm^−1^ and −28 cm^−1^, respectively.

GO is hydrophilic whereas graphene is hydrophobic,^45^ and the water contact angle on GO and rGO have been reported to cover a wide range depending on the reduction temperature.^46^ This is typically related to functional group variations, which may give rise to variations in the contact angles. The coatings presented herein contain rGO and APTES-rGO, and the hydrophilicity of rGO is intermediate between hydrophobic graphene and hydrophilic GO. Consequently, when rGO and APTES-rGO are present in the coating, the influence on the contact angle is not *a priori* obvious.

It is interesting to note from Table 2 that the presence of rGO and APTES-rGO in the coating gave rise to different effects on the contact angle. Comparing the samples, we see that the presence of rGO in the coating increased the contact angle, *i.e.*, the hydrophilicity decreased, whereas addition of APTES-rGO resulted in a lower contact angle, *i.e.*, the coating became more hydrophilic. The latter can be ascribed to the hydrophilic nature of the APTES-substituent relative to the functional groups present on the rGO surface.

**Table 2:** Contact angles without and with 5 wt% rGO and APTES-rGO present in the coating. Numbers are in deg

Addition to coating		
None	rGO	APTES-rGO
89.0	100.3	83.3

**Fig. 11:**
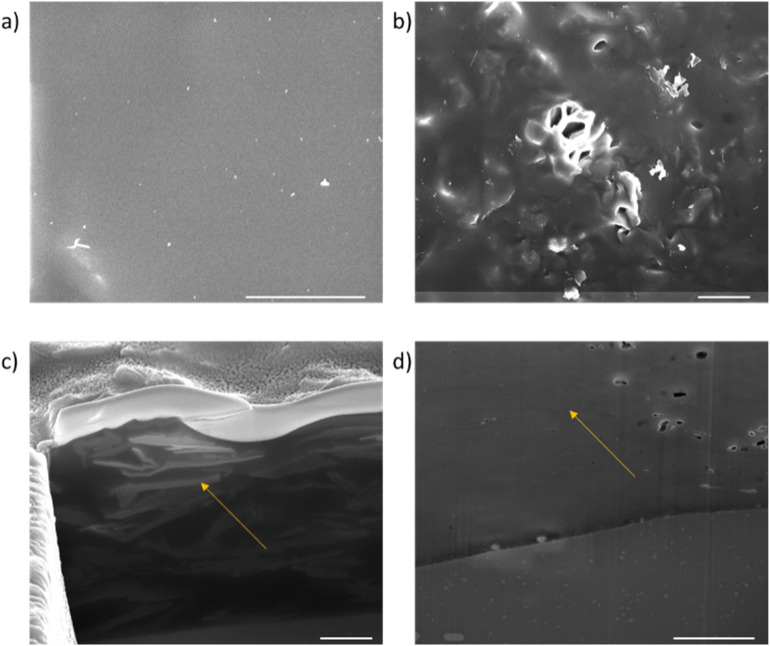
Plan SEM views of the coatings. (a) APTES-rGO and (b) rGO, cross sectional views of the coatings (c) APTES-rGO and (d) rGO. The scale bar is 5 μm.

FIB/SEM cross-sections were carried out on the coatings as highlighted in Fig. 11. The plan views shown in Fig. 11(a) and (b) show that for the rGO coating, the top surface is mostly polymer dominated. By contrast, texture from the embedded APTES-rGO can be seen on the surface of the APTES-rGO coating indicated a more homogeneous mixture. To further investigate the dispersion of the rGO flakes in the coating, FIB cross-sections were carried out. As shown by the arrow in Fig. 11(d), the rGO flakes agglomerate and lie in a random orientation as shown by the areas of light contrast (the sol gel matrix is dark in contrast). Voids can also be seen in the rGO sample. These voids have been thought to form around rGO flakes and are further evidence of a less homogenous mixture. Conversely, the APTES-rGO (seen in Fig. 11(c)) flakes are more evenly distributed in the matrix and appear to lie along the plane of the substrate. More images can be found in the ESI.†

Others have demonstrated the importance of GO orientation in various settings.^9–11^ We suggest that alignment can be used to tune sol–gel based coating properties, and we propose that diffusion barrier modulation properties is particularly relevant.

## Conclusions

Reduced graphene oxide (rGO) and APTES functionalised reduced graphene oxide (APTES-rGO) have been included in composite coatings prepared by sol–gel technology. Functionalisation occurred through the formation of an amide bond and GO reduction, and the 2D sheets were retained. A key finding is that the rGO flakes agglomerate and lie in a random orientation in the coating, whereas the APTES-rGO flakes are more evenly distributed in the matrix and appear to lie along the plane of the substrate. Finally, incorporation of APTES-rGO in the coating increased the hydrophilicity, whereas rGO increased the hydrophobicity.

## Author contributions

Conceptualization: BDB, AMB, ÖA, HA, KT. Formal analysis: BDB, TD, ITJ, PAC, AF, JY, AMB, ÖA, HA, NE, AK, KT. Funding acquisition: HA, BDB, AMB, ÖA, KT. Investigation: BDB, TD, ITJ, PAC, AF, ÖA, NE, AK, KT. Project administration: HA, ÖA, AMB, KT. Validation: BDB, TD, ITJ, PAC, AF, JY, AMB, ÖA, HA, NE, AK, KT visualization: BDB, ITJ, PAC, JY, MG, AF, AMB, ÖA, HA, NE, AK, KT writing – original draft: BDB, ITJ, PAC, AF, JY, AMB, ÖA, HA, NE, AK, KT writing – review & editing: BDB, TD, ITJ, PAC, AF, JY, AMB, ÖA, HA, NE, AK, KT. All authors have agreed to the final version of the manuscript.

## Conflicts of interest

There are no conflicts to declare.

## Supplementary Material

NA-006-D3NA01057K-s001
