# Supplementary material for: Orientation of reduced graphene oxide in composite coatings
Source: Nanoscale Adv. 2024 Mar 11;6(8):2088–95. doi: 10.1039/d3na01057k (PMC11019492; doi:10.1039/d3na01057k)
Supplement: NA-006-D3NA01057K-s001 [file NA-006-D3NA01057K-s001.pdf]

Electronic Supplementary Material (ESI) for Nanoscale Advances.
This journal is © The Royal Society of Chemistry 2024

Supporting information

Orientation of reduced graphene oxide in composite coatings

Knut Thorshaug†[a], Terje Didriksen[a], Ingvild Thue Jensen[a], Patricia Almeida Carvalho[a], Juan Yang[a], Matthieu Grandcolas[a], Alain Ferber[b], Andy M. Booth[c], Özlem Ağaç[d], Hüseyin Alagöz[d], Nursev Erdoğan[e], Anıl Kuban[e], and Branson D. Belle[a]

[a]SINTEF Industry, Forskningsveien 1, NO-0373 Oslo, Norway

[b]SINTEF Digital, Forskningsveien 1, NO-0373 Oslo, Norway

[c]SINTEF Ocean, Brattørkaia 17C, NO-7010 Trondheim, Norway

[d]Nanografi Nanotechnology AS, ODTÜ Teknokent No:13/1-1 06531 Çankaya, Ankara, Turkey

[e]Turkish Aerospace, Functional Coatings & Transparencies Technology Centre, Ankara, Turkey

†Corresponding author: knut.thorshaug@sintef.no

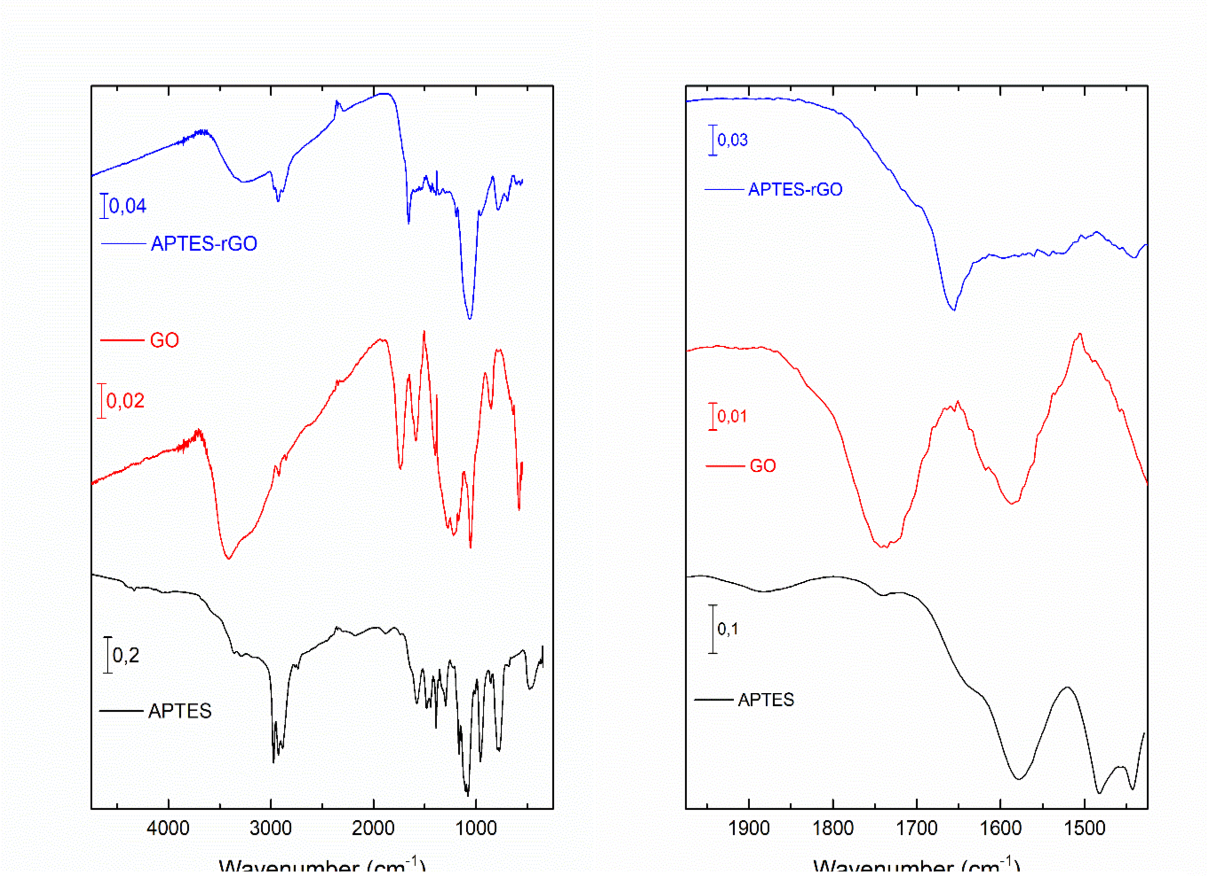

Figure S1. FTIR spectra for the starting materials APTES (bottom, black) and GO (middle, red), and the product APTES-rGO (upper, blue).

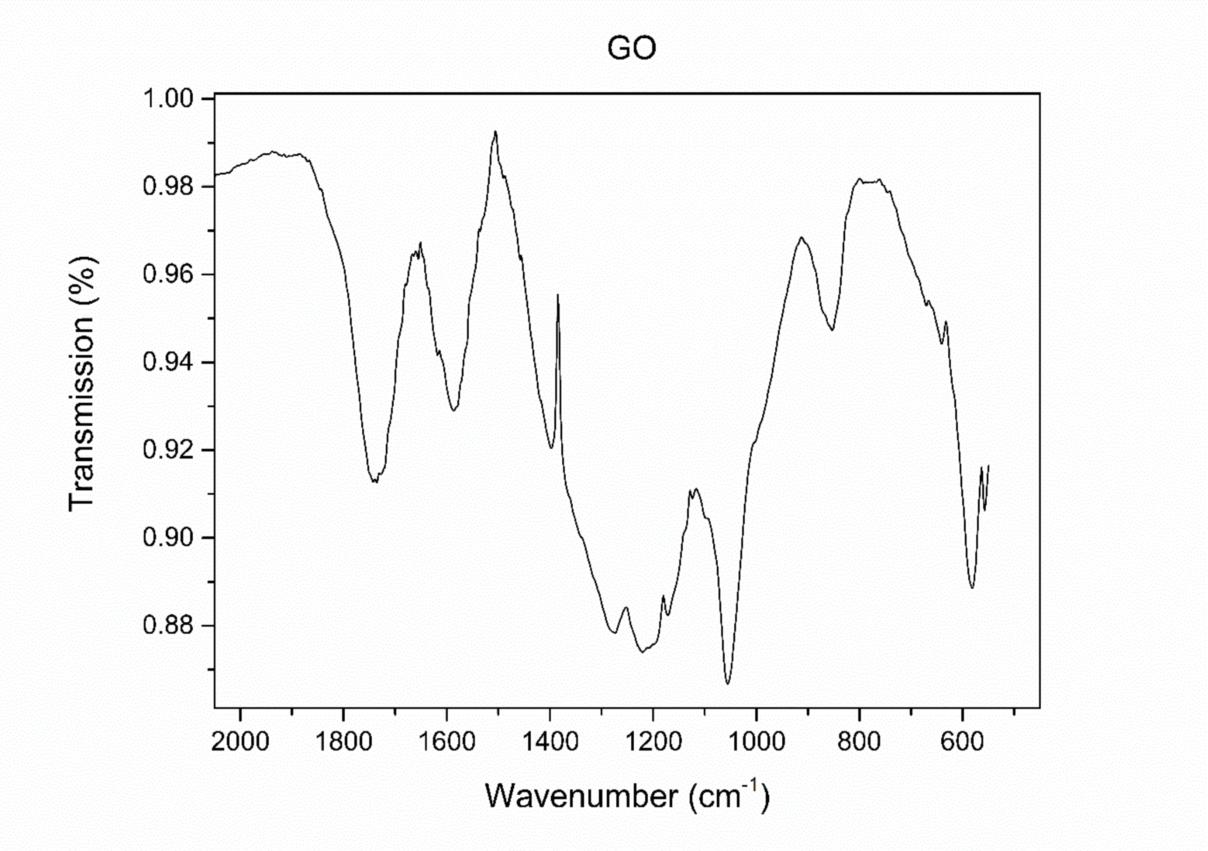

Figure S 2. FTIR spectrum of GO.

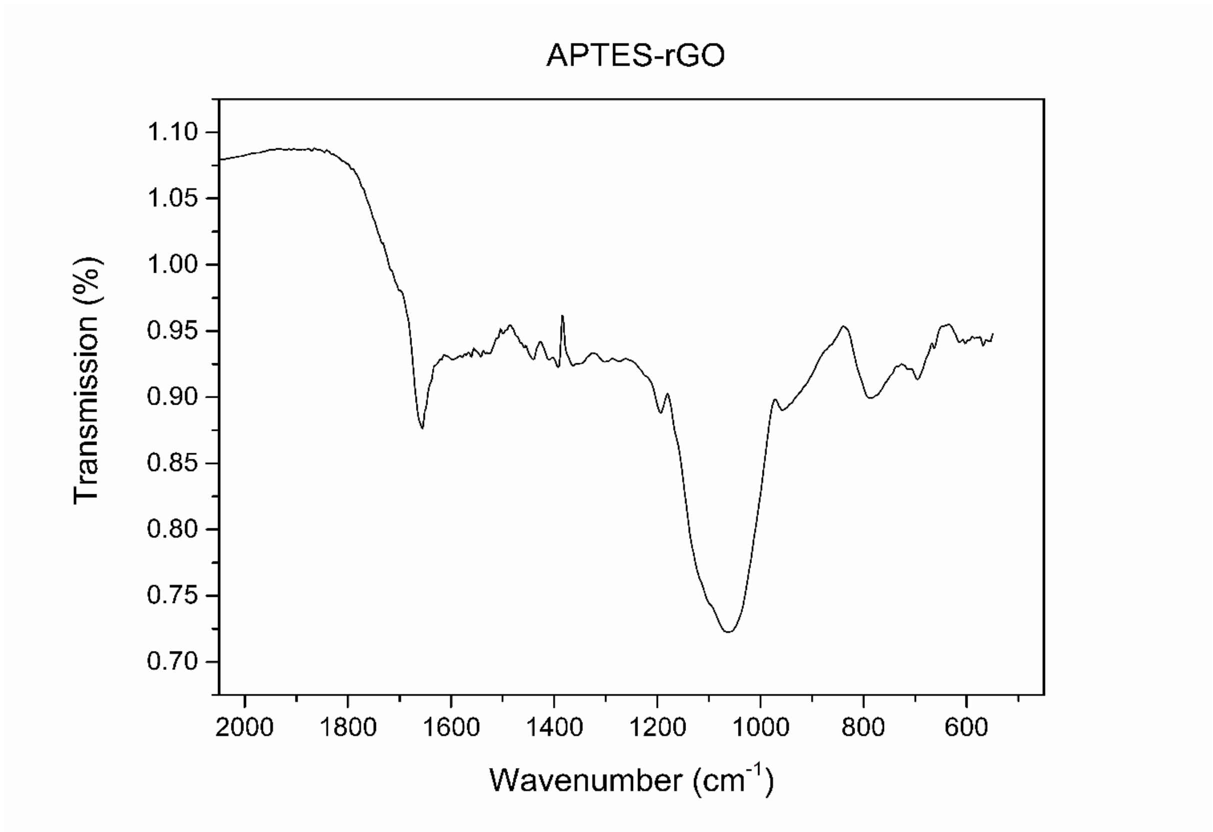

Figure S 3. FTIR spectrum of APTES-rGO.

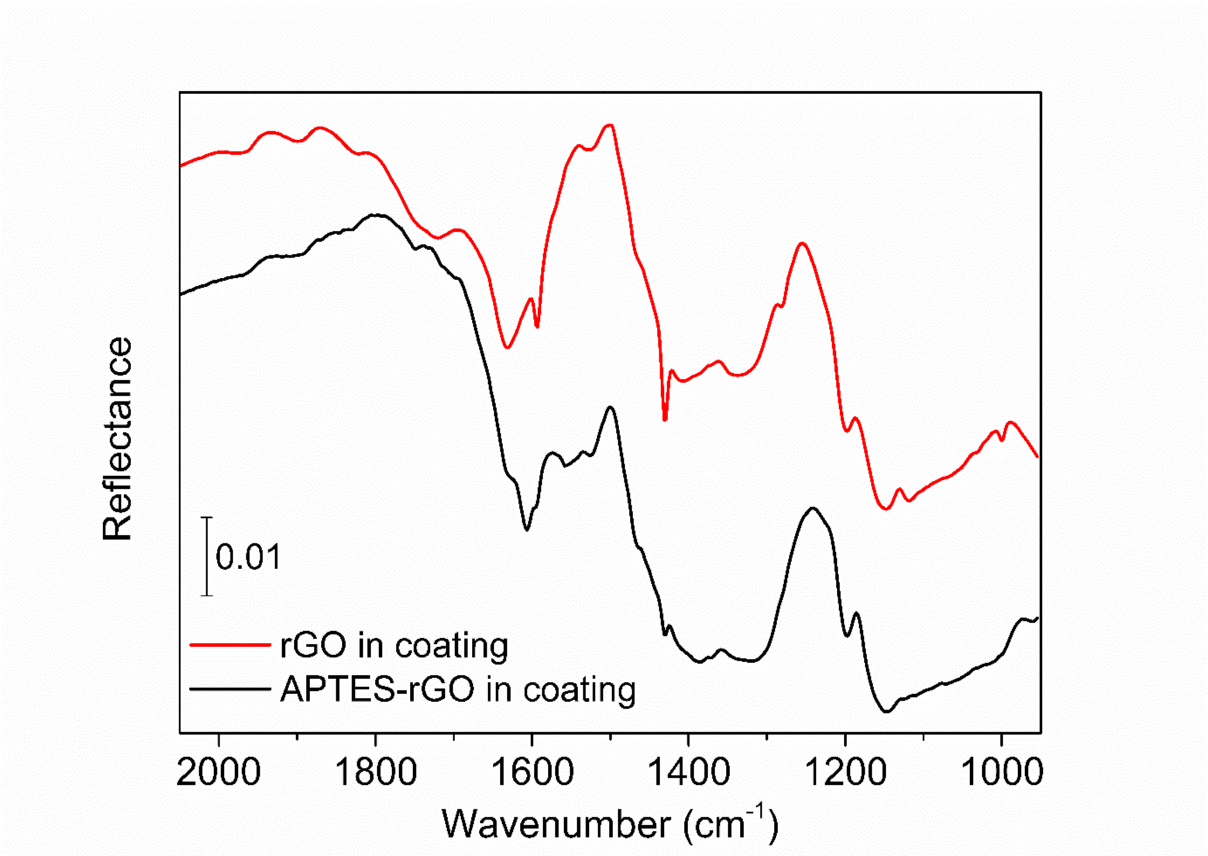

Figure S 4. FTIR spectra of coating containing APTES-rGO (black line) and not-containing APTES-rGO (red line).

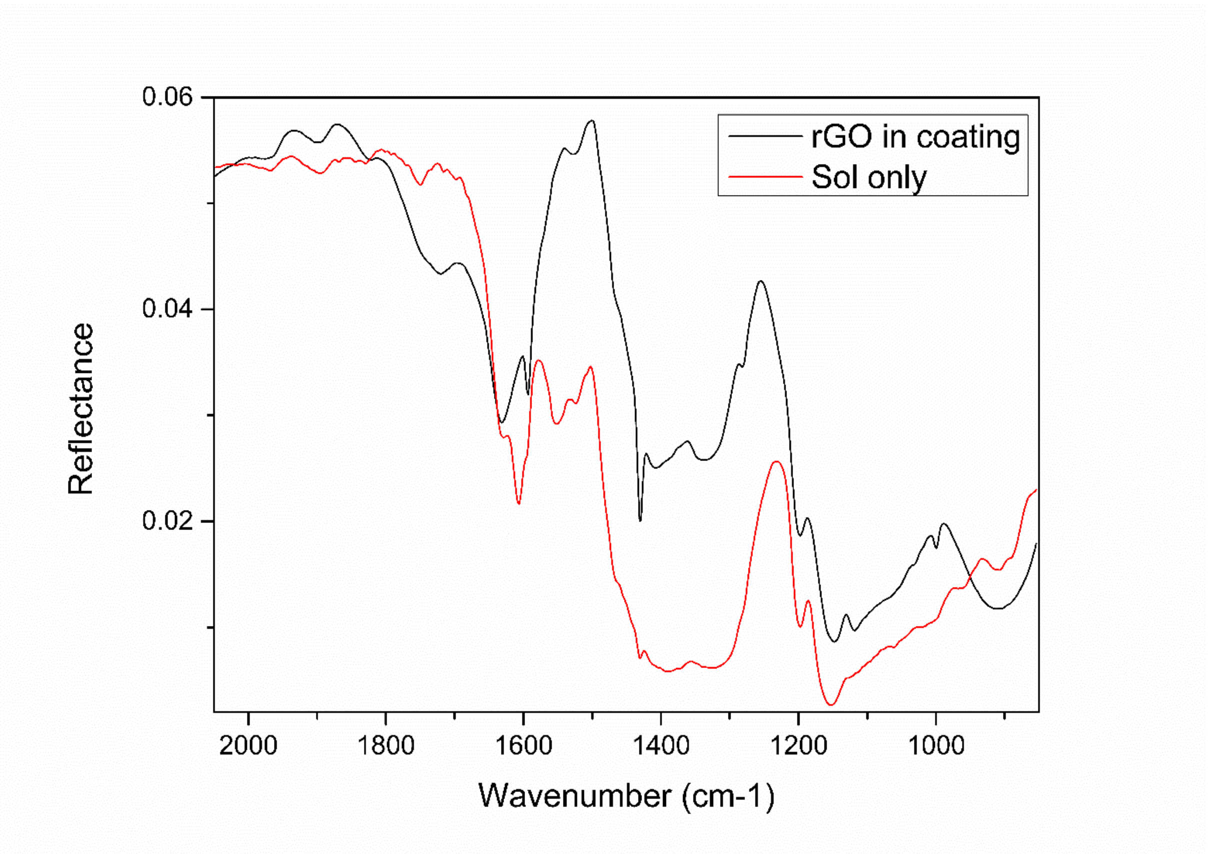

Figure S 5. FTIR spectra of coating containing rGO (black line) and not-containing APTES-rGO (red line).

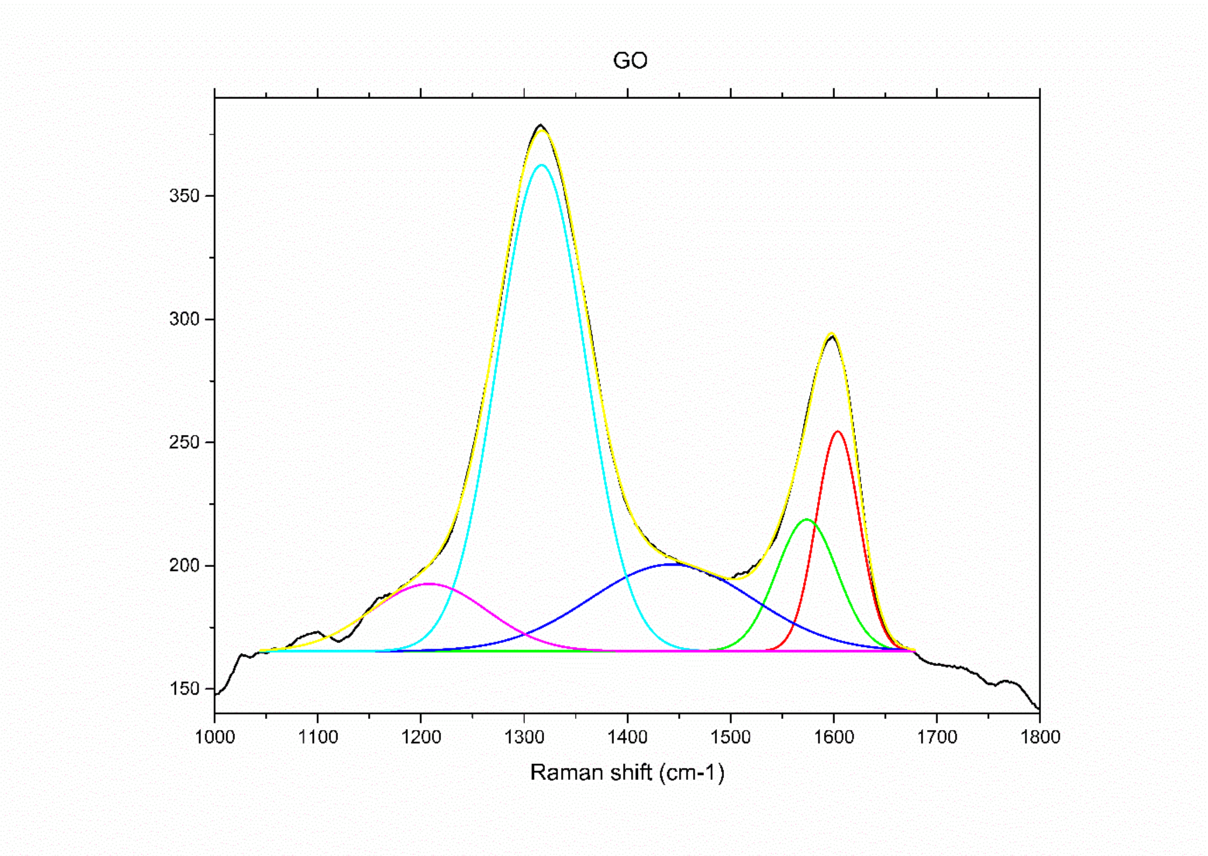

Figure S 6. Curve-fits to Raman spectra for GO. Gauss fits.

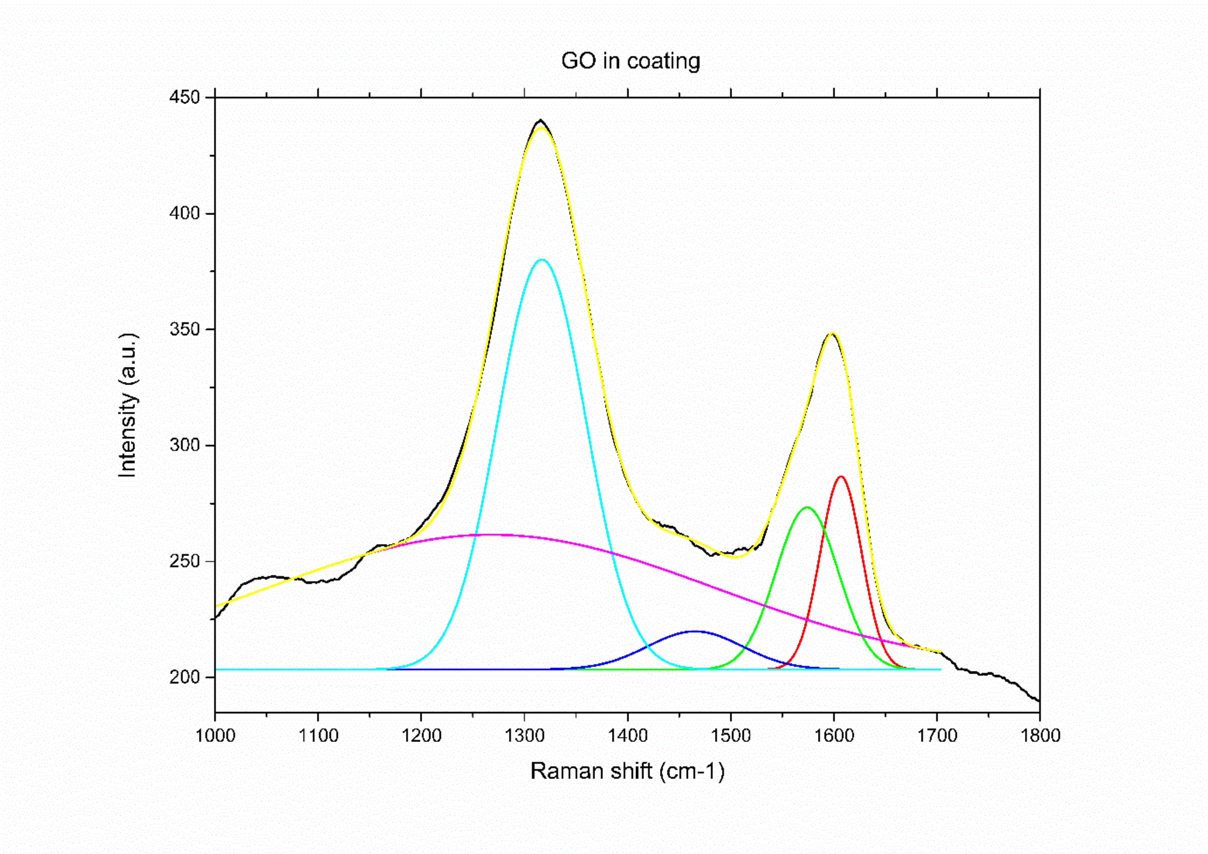

Figure S 7. Curve-fits to Raman spectra for rGO in coating. Gauss fits.

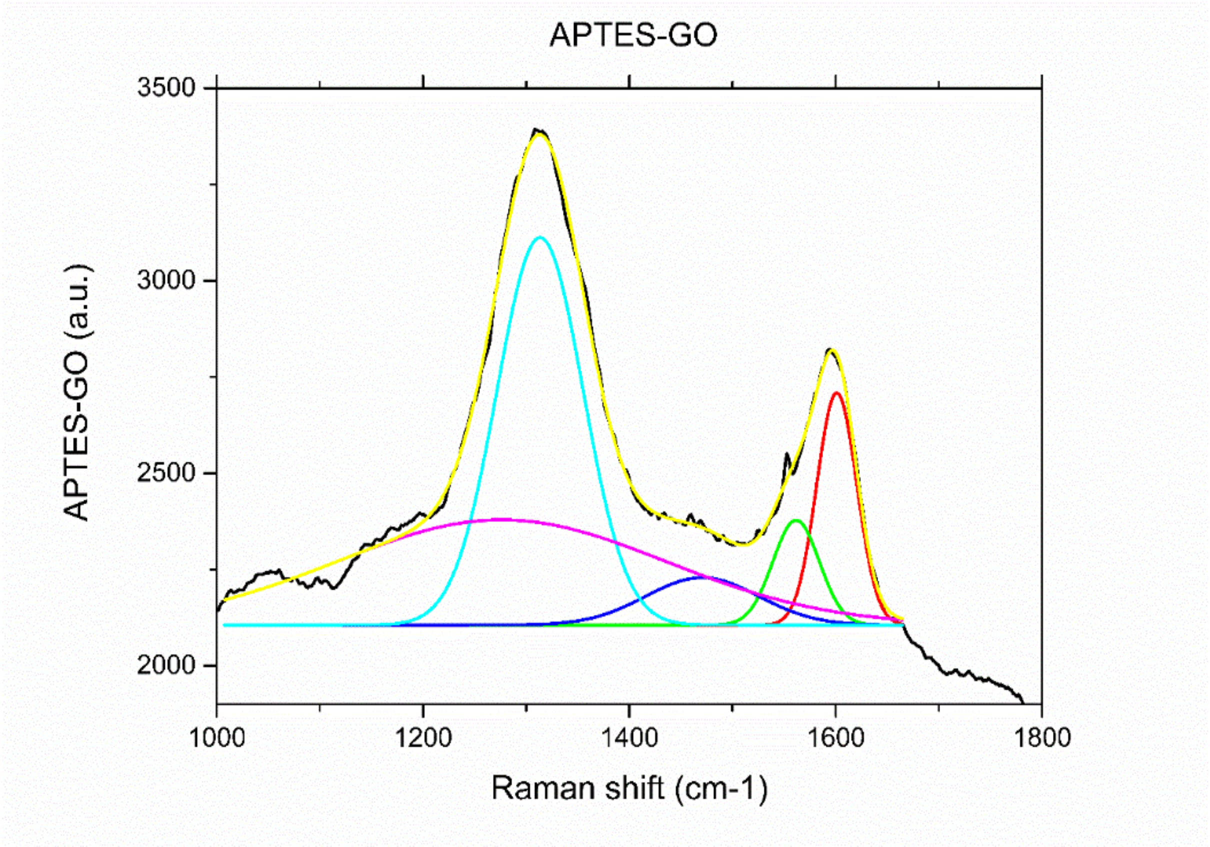

Figure S 8. Curve-fits to Raman spectra for APTES-rGO. Gauss fits.

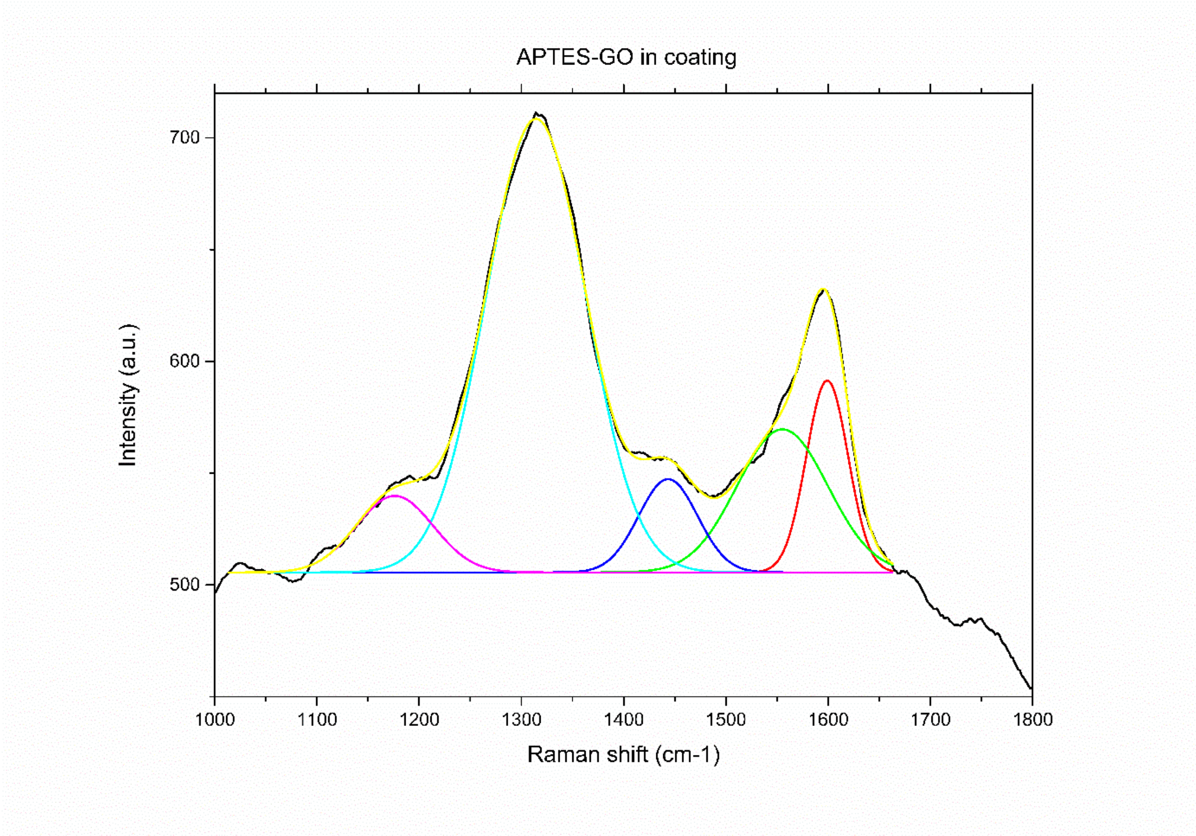

Figure S 9.Curve-fits to Raman spectra for APTES-rGO in coating. Gauss fits.

Table S 1. Band position (xc) and full width half at half maximum (FWHM) from fitting of five bands to the experimental Raman spectra. We used Gauss fits.

	**D***		**D**		**D''**		**G**		**D'**	
	xc	FWHM	xc	FWHM	xc	FWHM	xc	FWHM	Xc	FWHM
	[cm^{-1}]	[cm^{-1}]	[cm^{-1}]	[cm^{-1}]	[cm^{-1}]	[cm^{-1}]	[cm^{-1}]	[cm^{-1}]	[cm^{-1}]	[cm^{-1}]
**GO**	1208	132	1317	100	1443	185	1574	69	1604	49
**APTES-rGO**	1276	374	1313	98	1471	129	1562	53	1601	46
**rGO in coating**	1160*		1317	101	1465	109	1574	71	1607	47
**APTES-rGO in coating**	1176	89	1314	116	1443	68	1555	104	1599	48

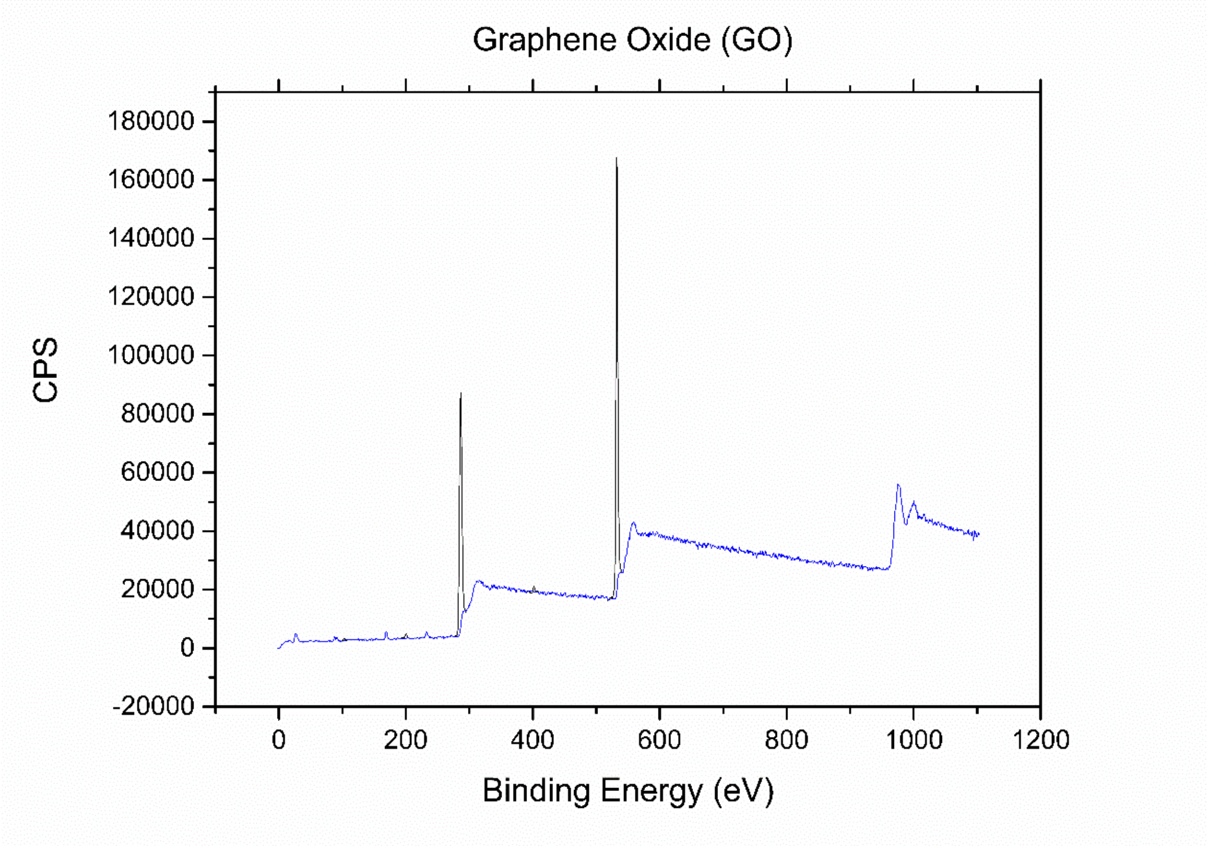

Figure S 10. XPS survey GO.

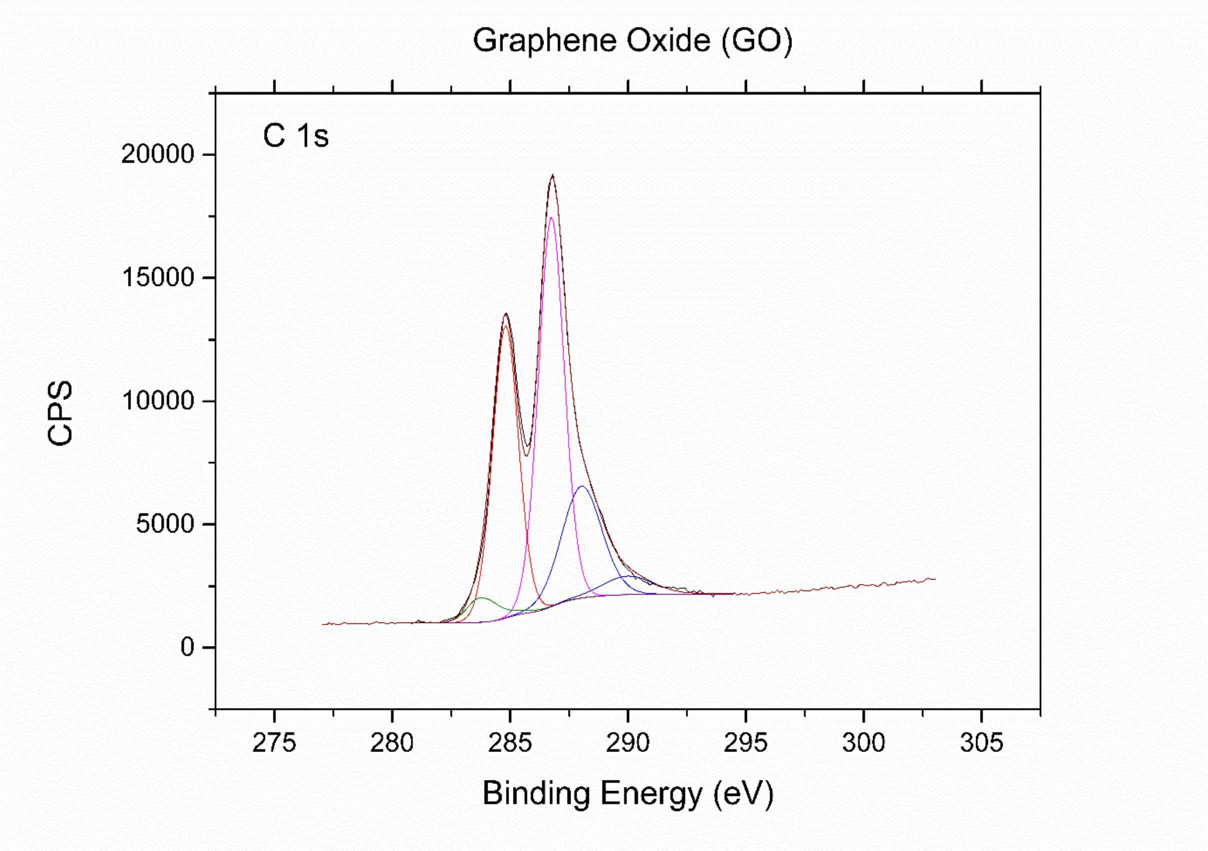

Figure S 11. GO C1s

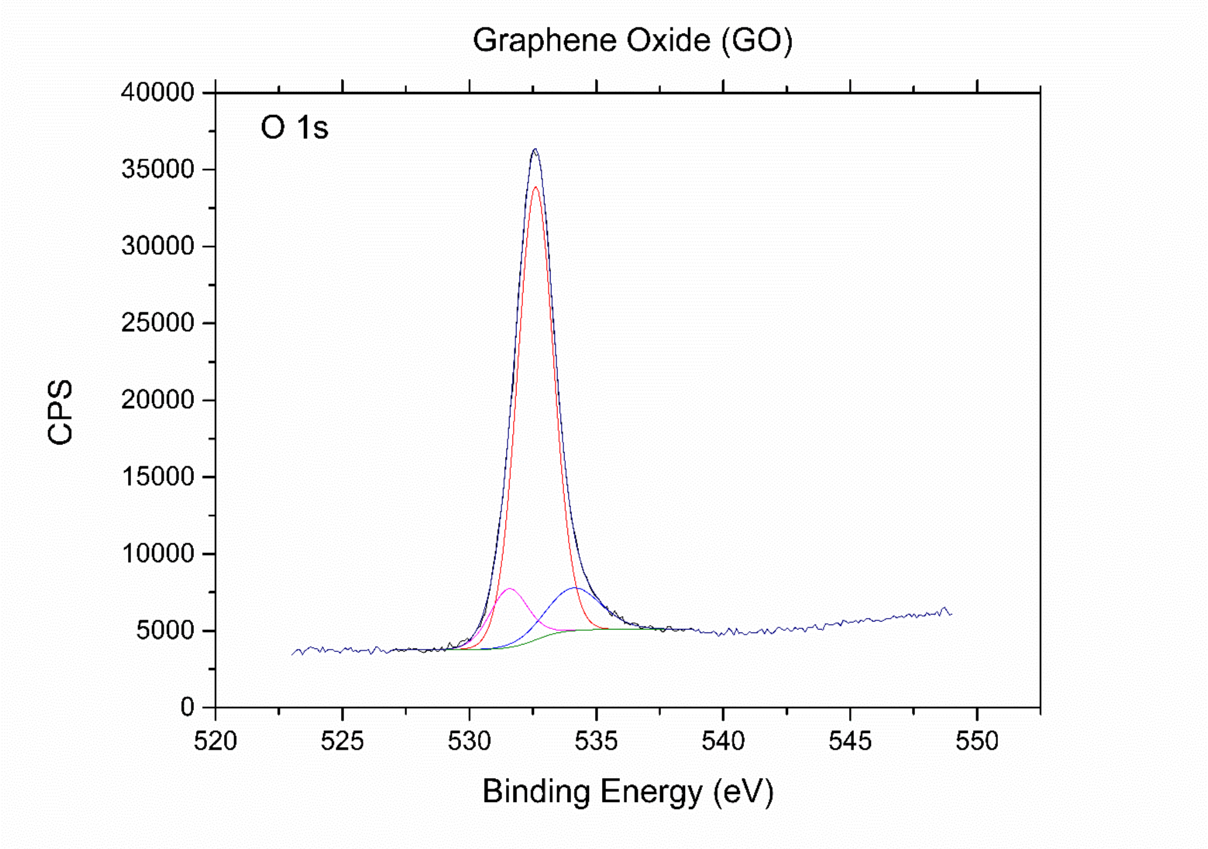

Figure S 12. GO O1s.

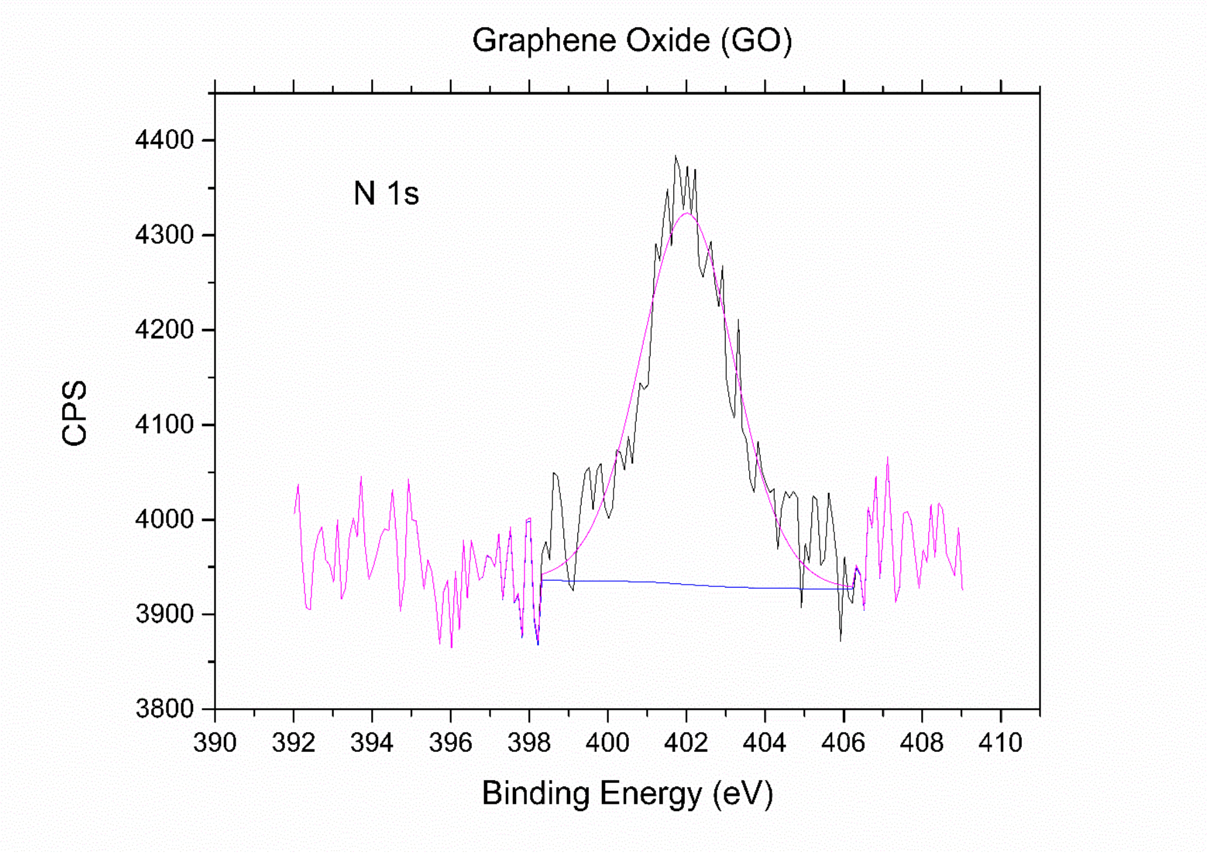

Figure S 13. GO N1s.

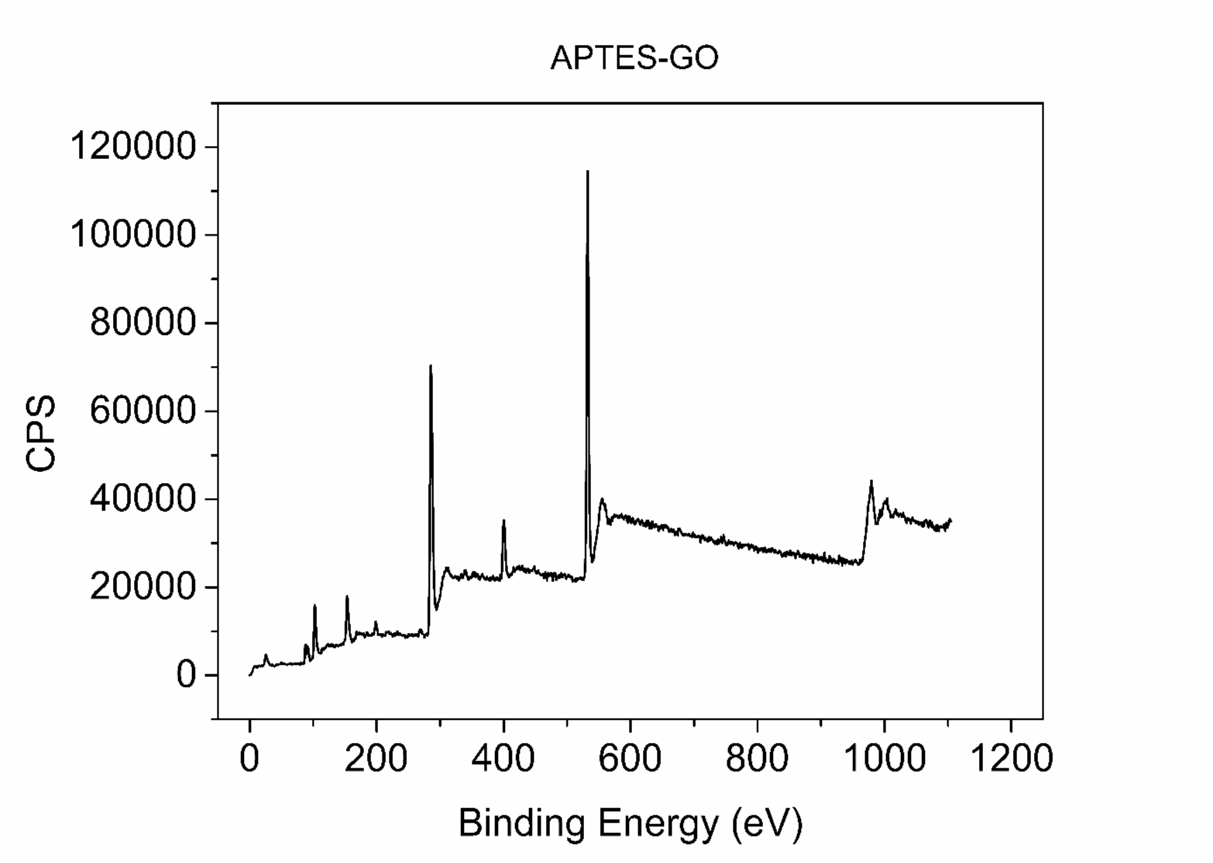

Figure S 14. XPS survey APTES-rGO.

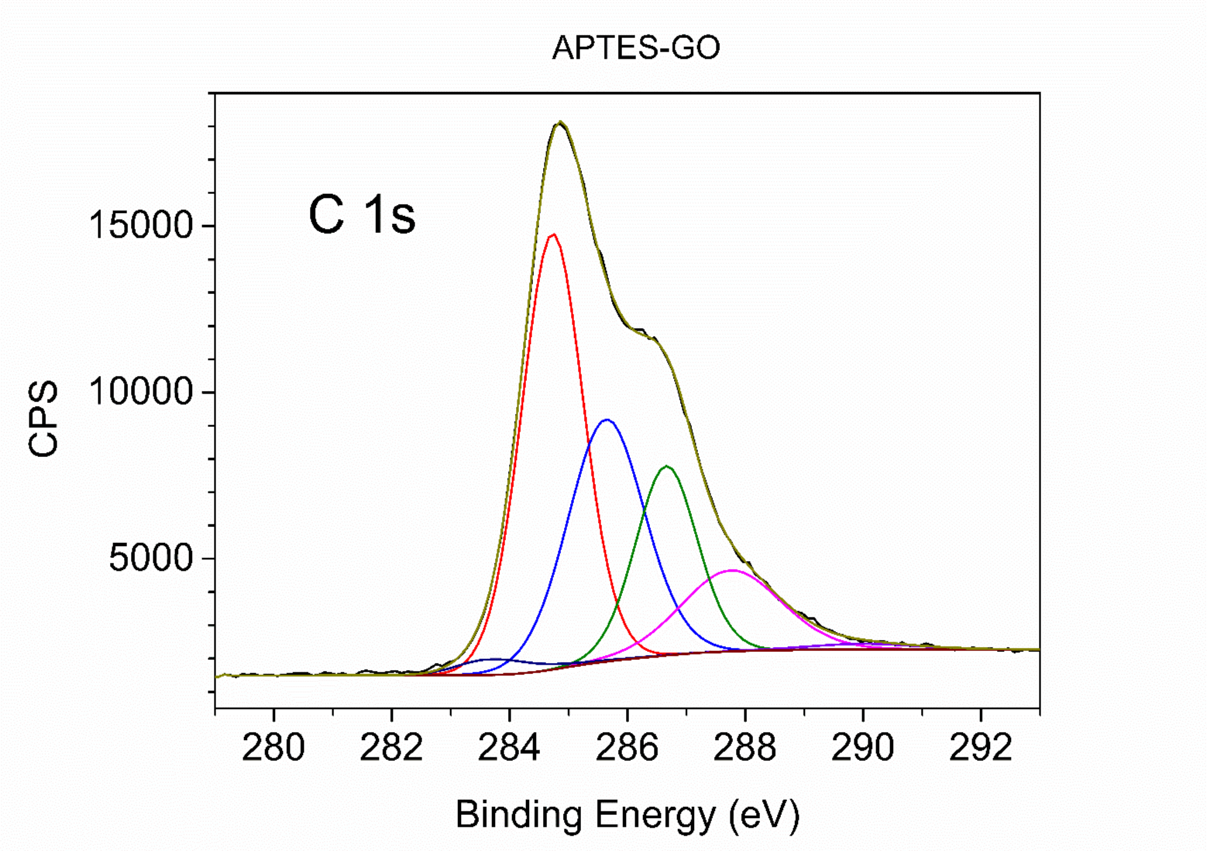

Figure S 15. APTES-rGO C1s.

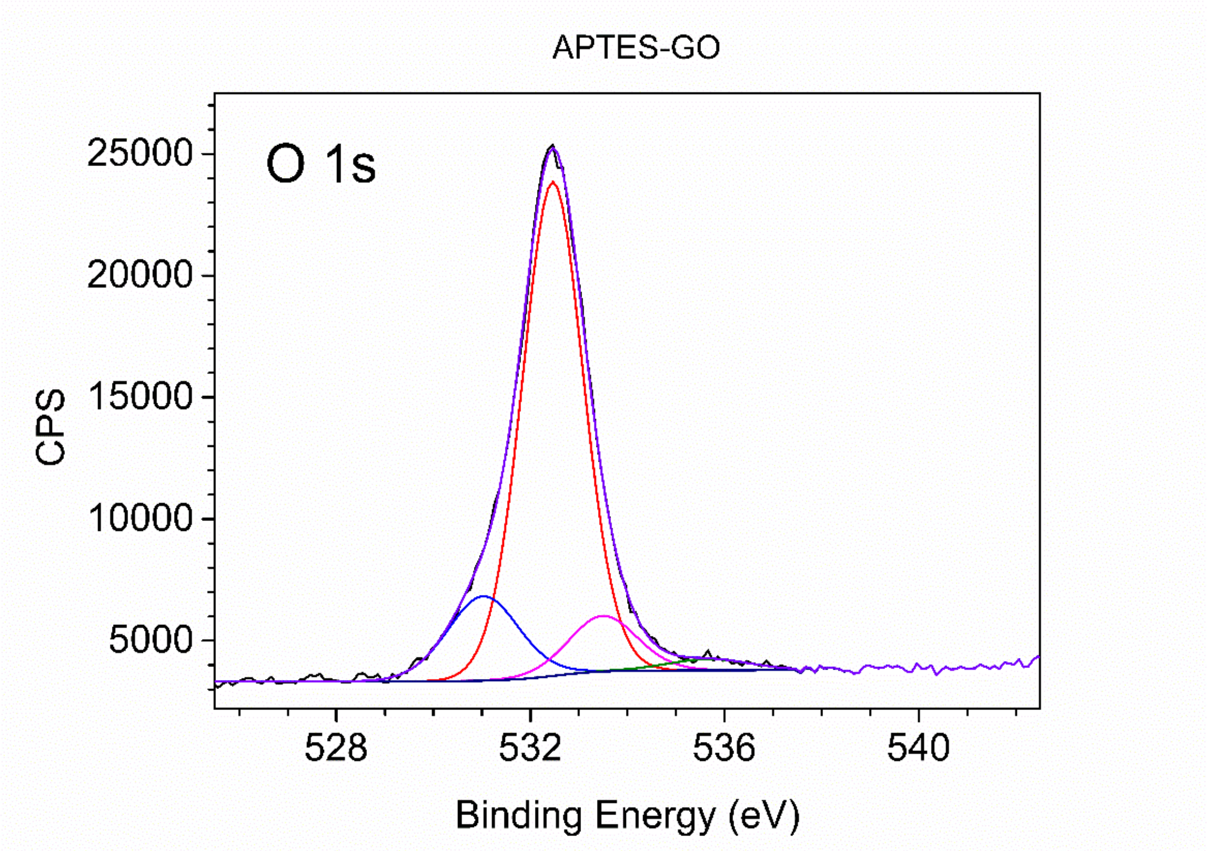

Figure S 16. APTES-rGO O1s.

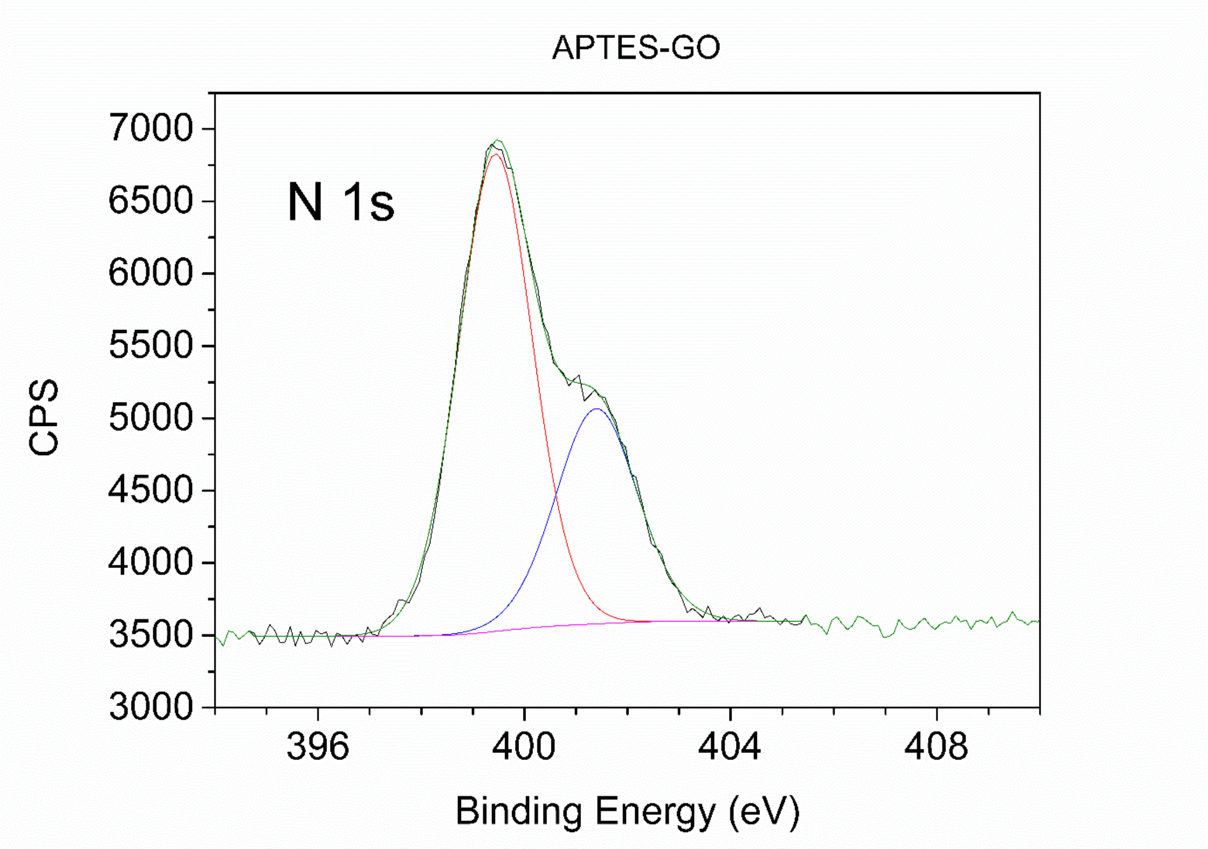

Figure S 17. APTES-rGO N1s.

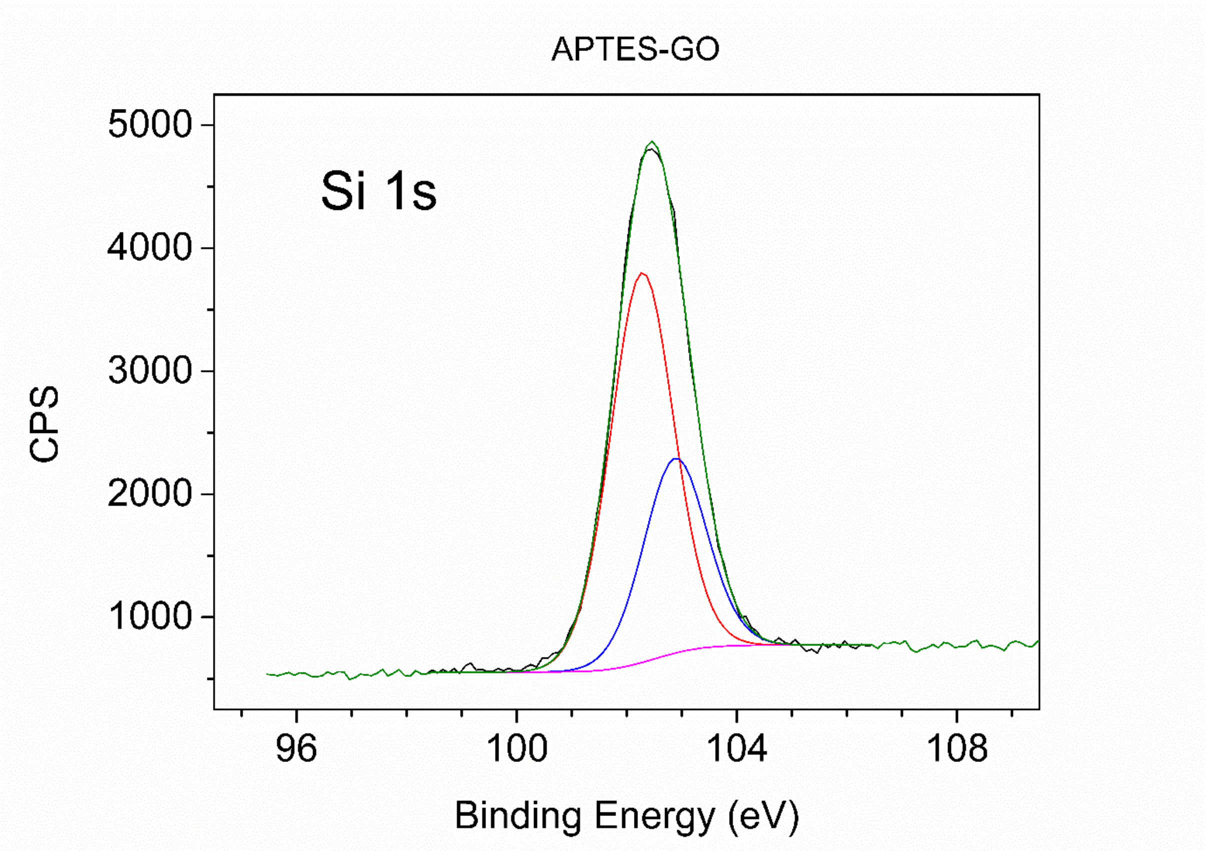

Figure S 18. APTES-rGO Si1s.

Images

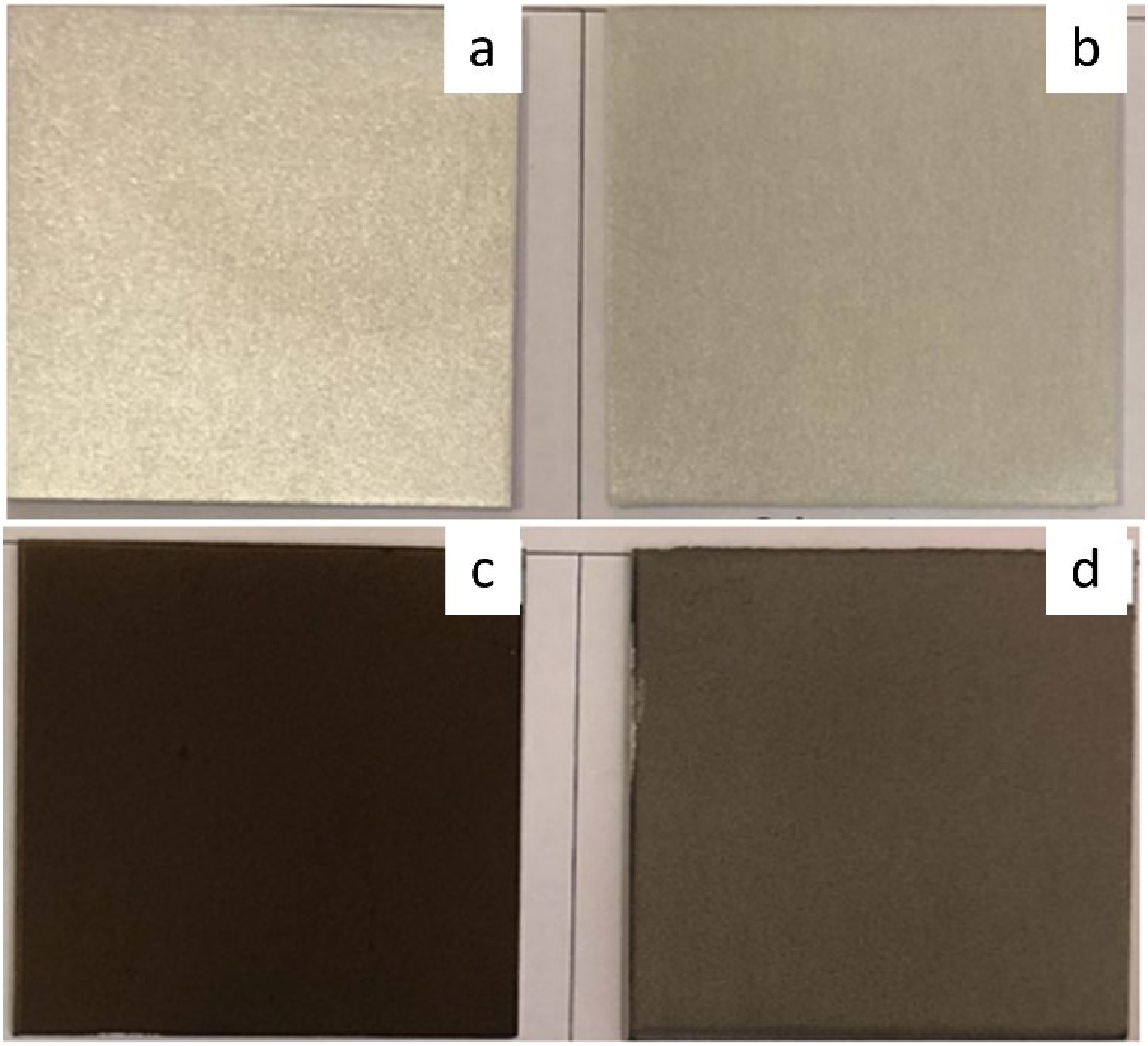

Figure S 19. Coatings on Al 2024 T-3. (a) no coating, (b) sol-gel only, (c) rGO in sol-gel, (d) APTES-rGO in sol-gel. Sample size 5×5 cm.

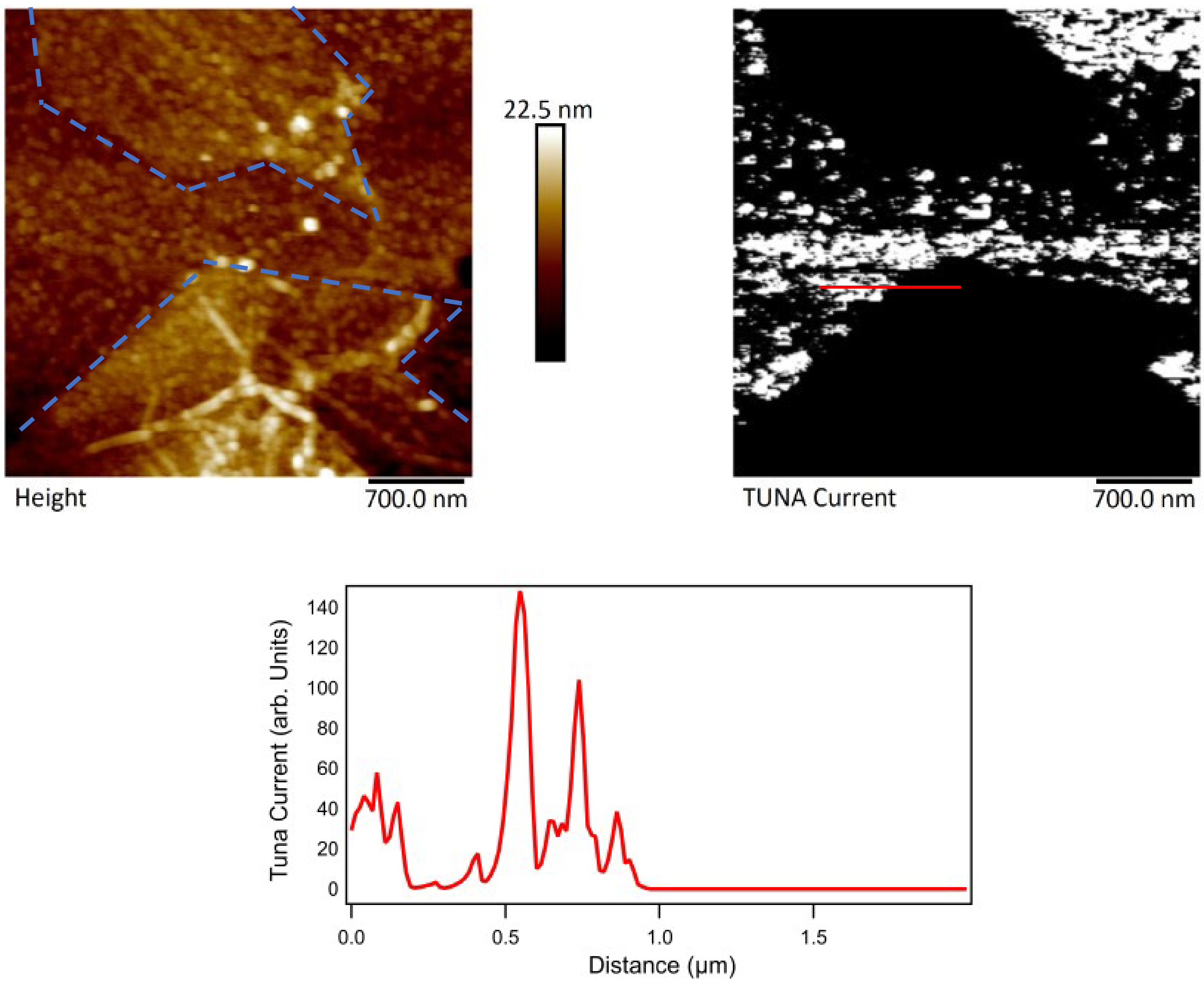

Figure S 20. GO was deposited on Au coated Silicon. The flake is outlined by the blue dashed line. Peak force TUNA was used to map electrical conductivity of the flakes using a PtIr tip. As seen in the TUNA current map. A section from the current map is also presented which shows no conductivity over the flake.

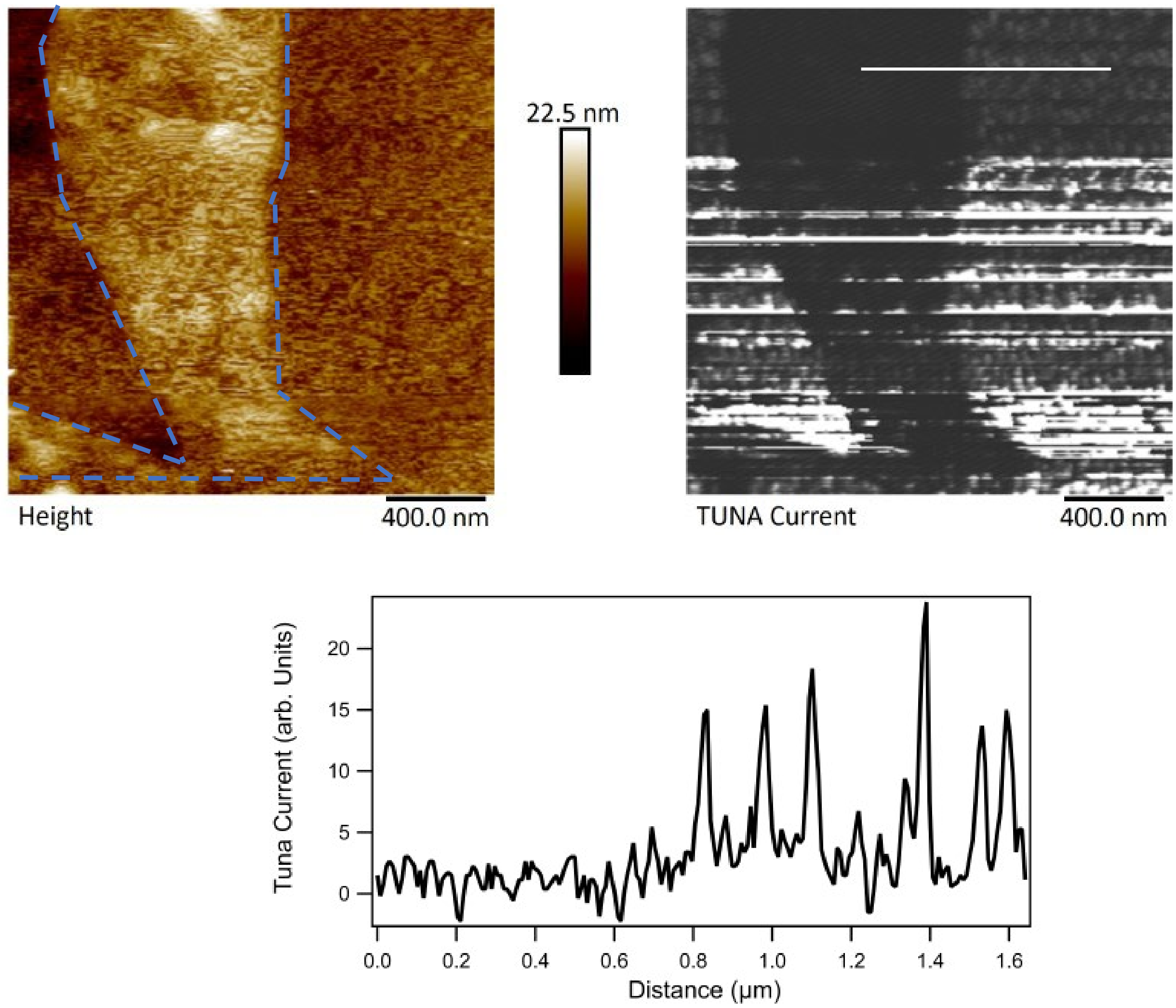

Figure S 21. APTES-rGO was deposited on Au coated Silicon. The flake is outlined by the blue dashed line. Peak force TUNA was used to map electrical conductivity of the flakes. As seen in the TUNA current map and section below, the APTES-rGO flake has limited conductivity which is increased in comparison to the GO flake which suggests some reduction has taken place in the APTES functionalisation process.

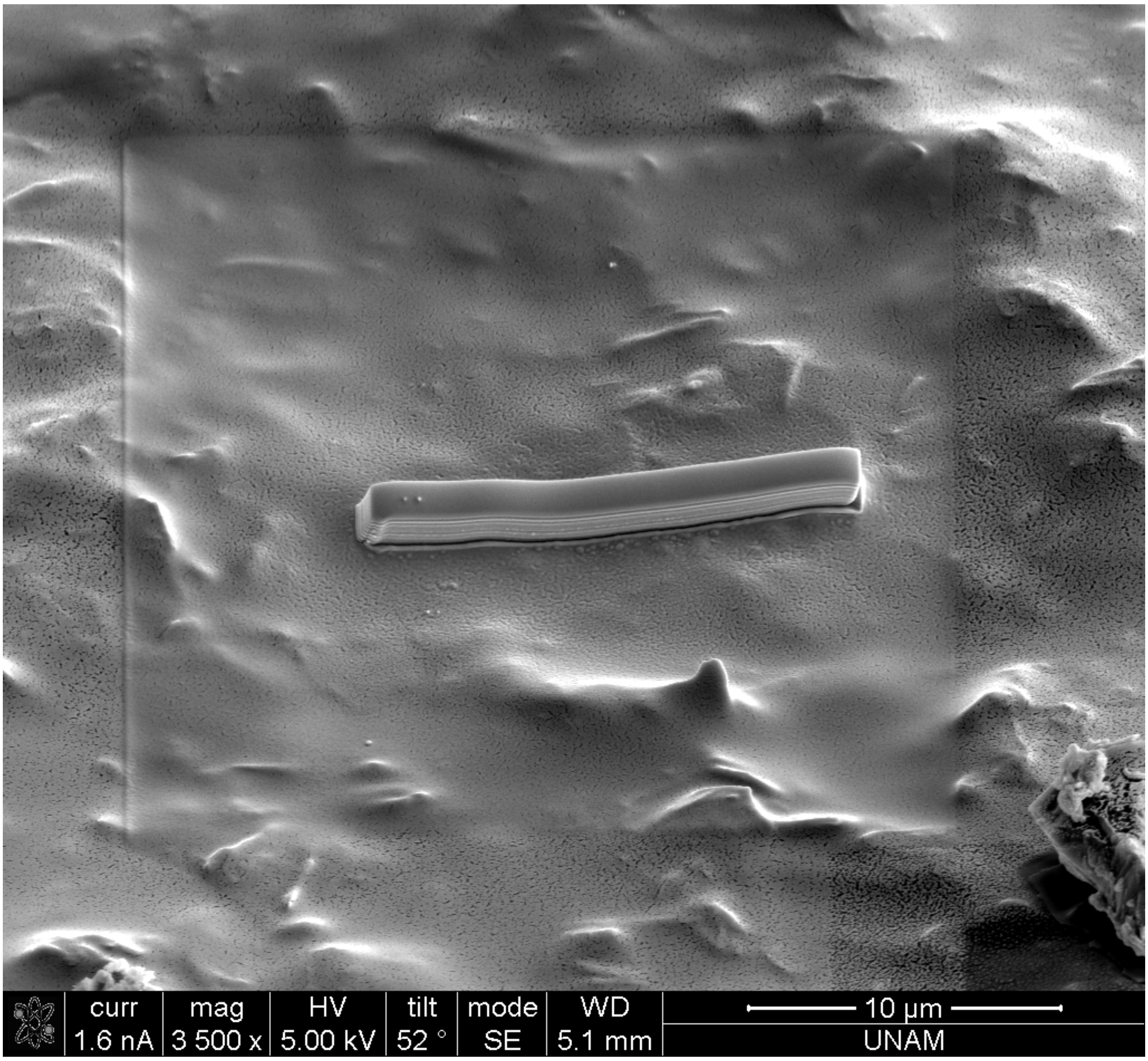

Figure S 22. SEM image of 5% APTES-rGO in coating.

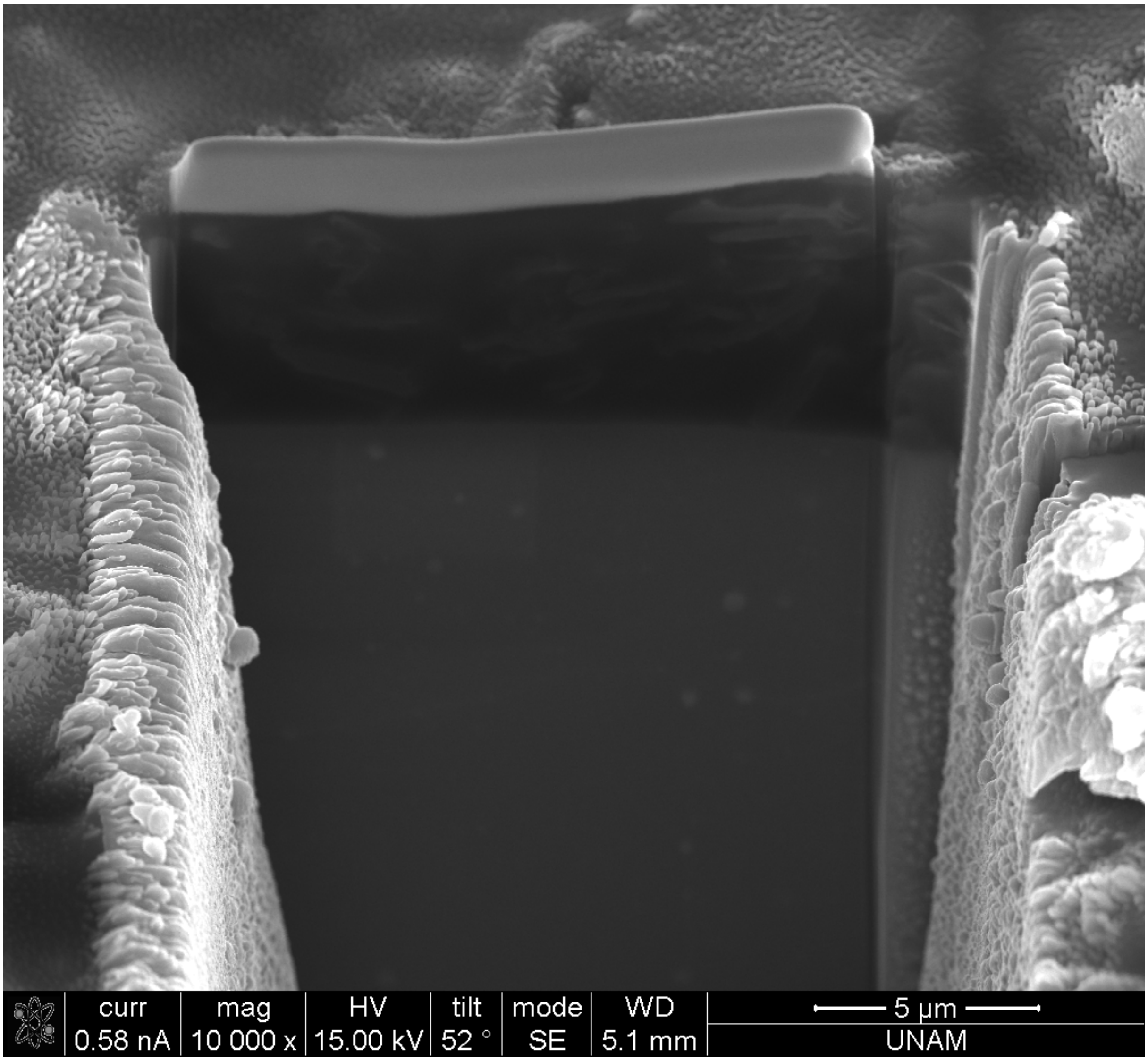

Figure S 23. SEM image of 5% APTES-rGO in coating.

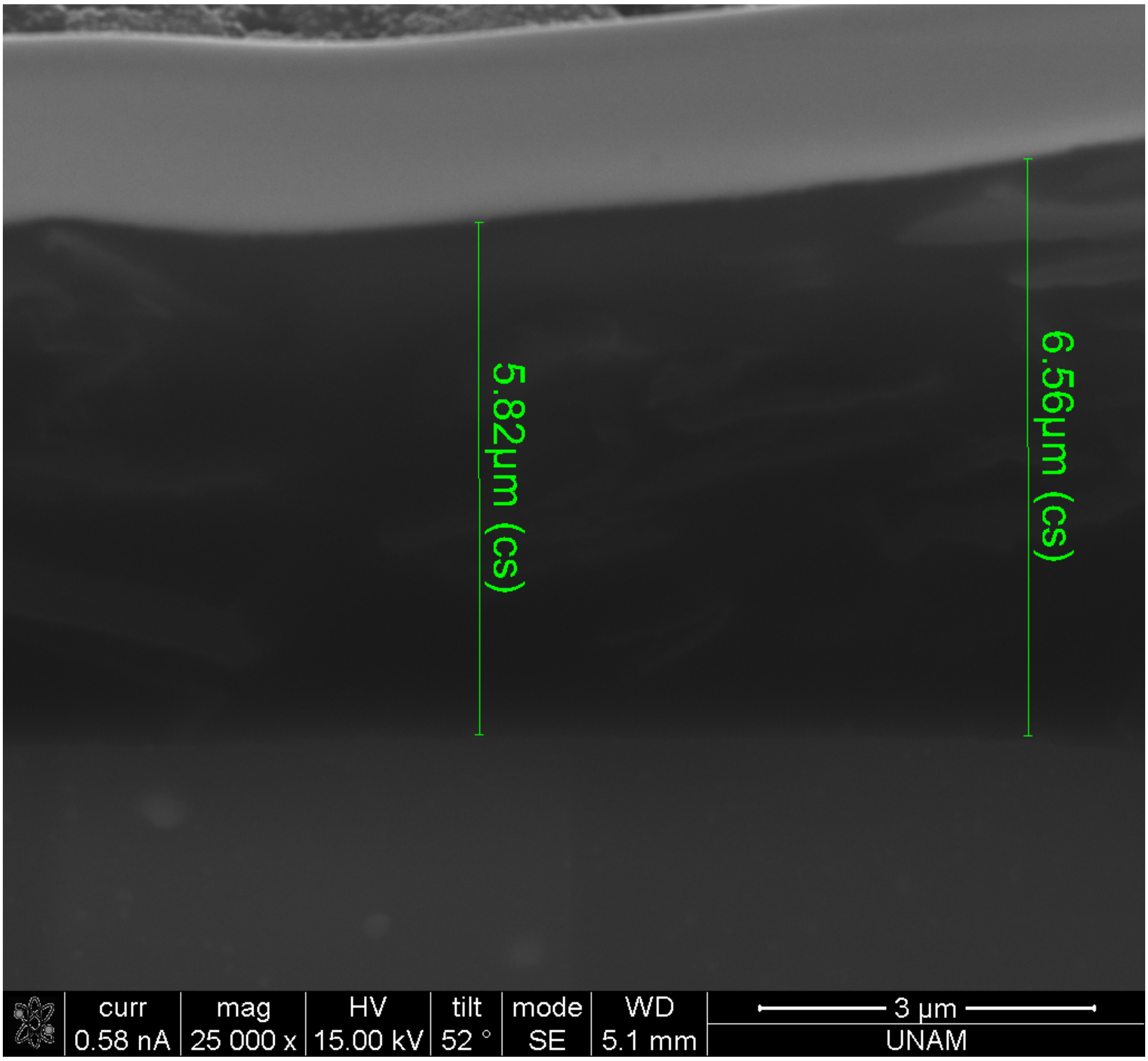
5.82µm (cs)
6.56µm (cs)
curr
0.58 nA
mag
25 000 x
HV
15.00 kV
tilt
52 °
mode
SE
WD
5.1 mm
3 µm
UNAM

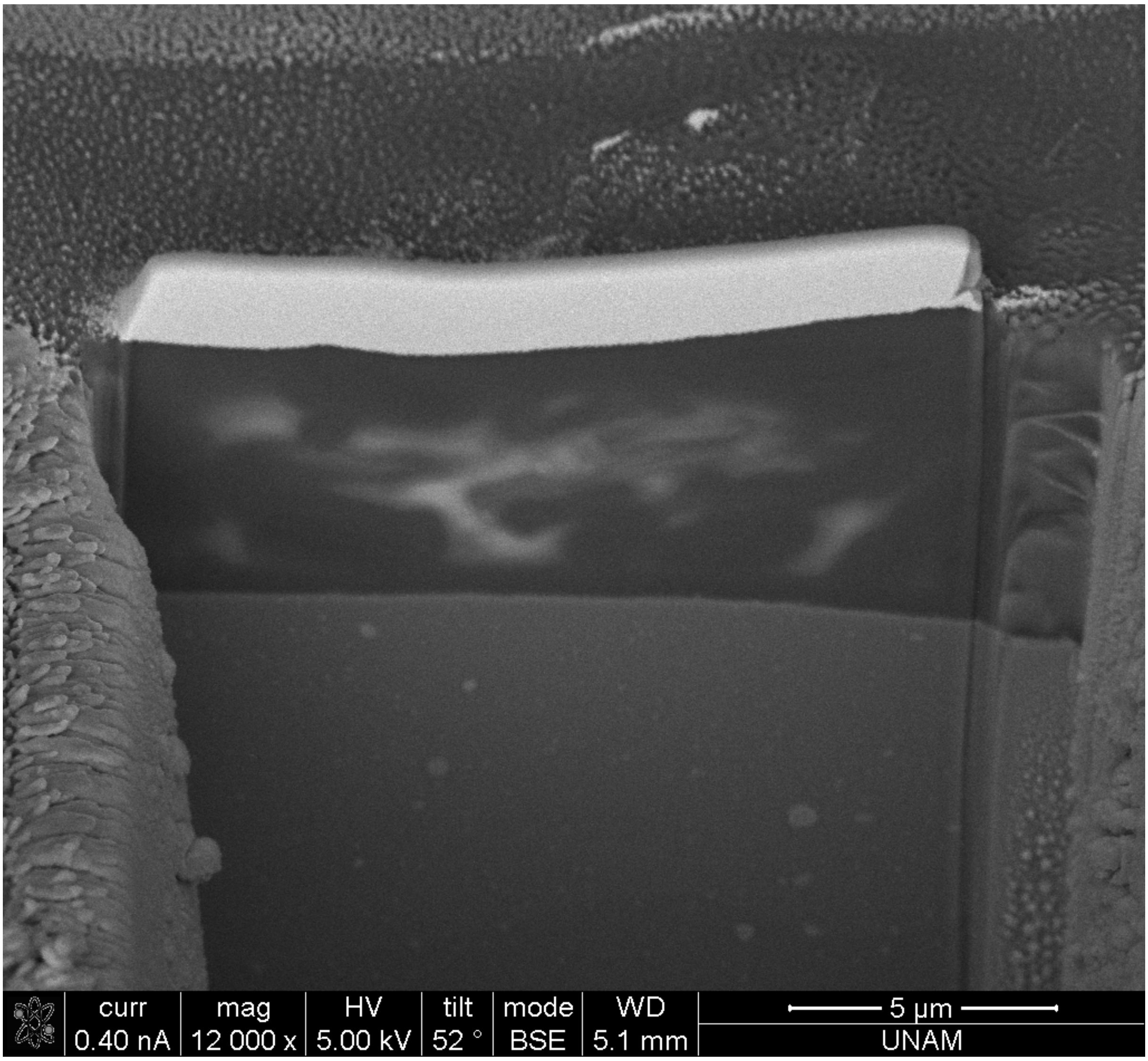

Figure S 24. SEM image of 5% APTES-rGO in coating.

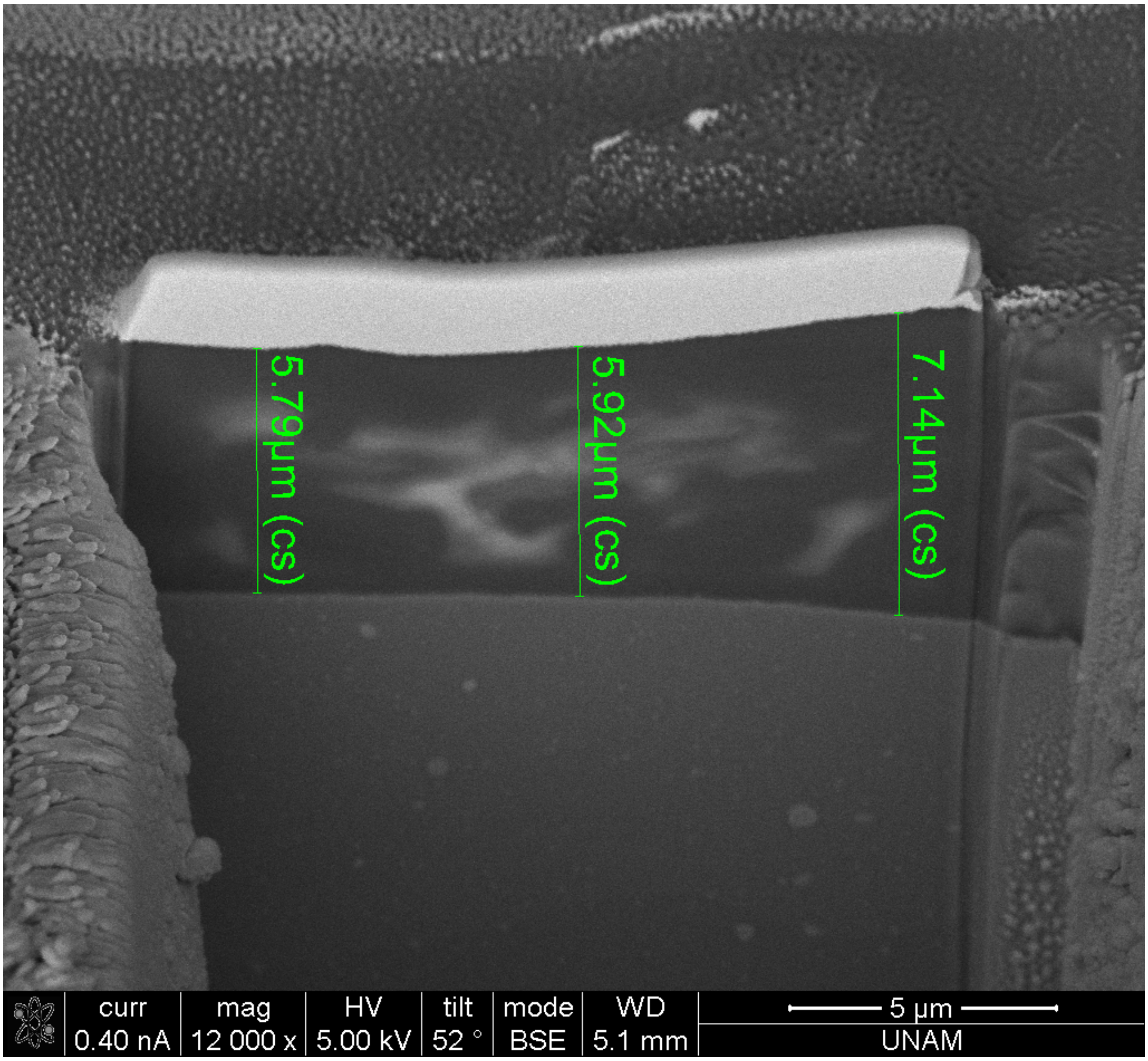

Figure S 25. SEM image of 5% APTES-rGO in coating.

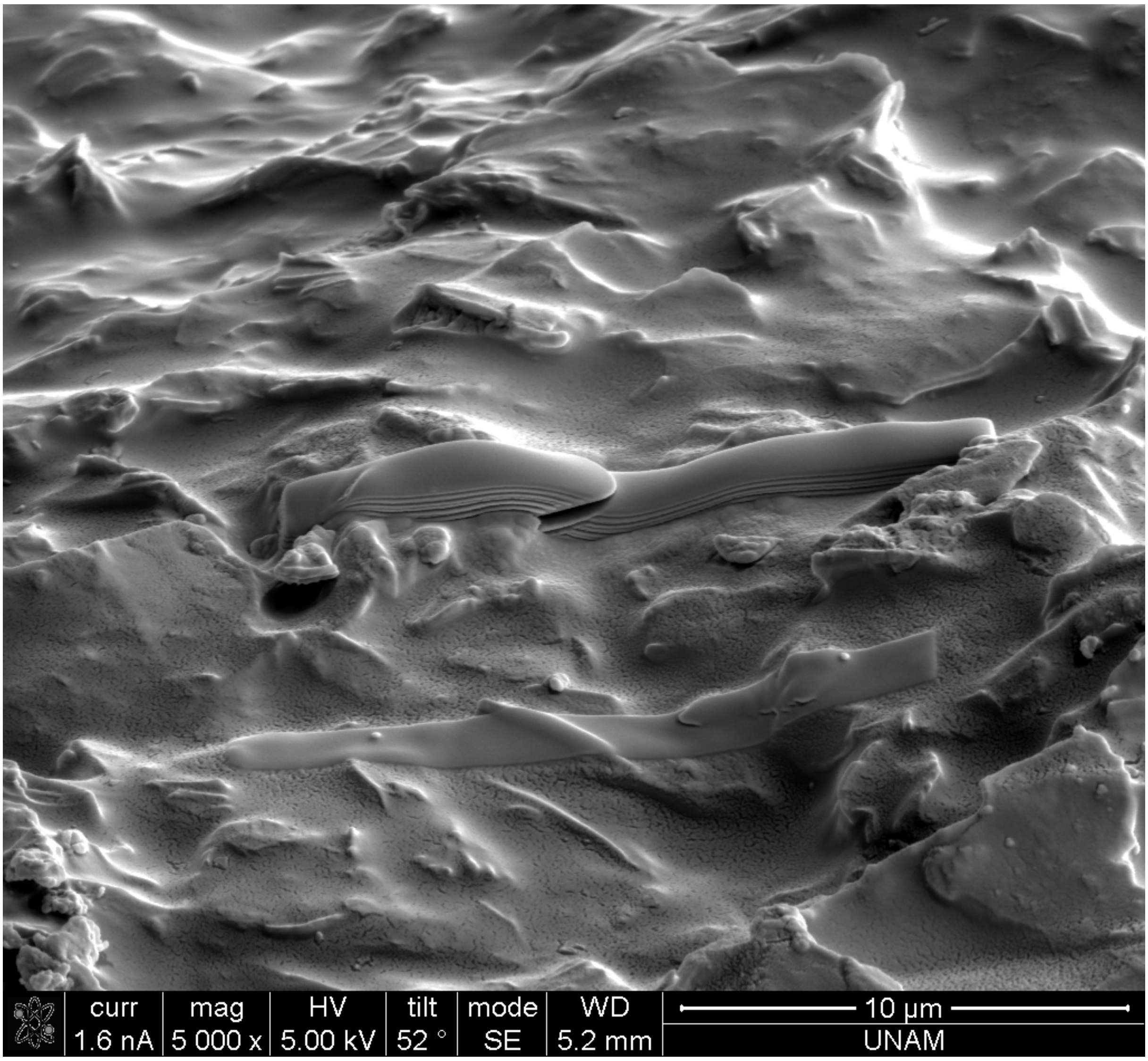

Figure S 26. SEM image of 10% APTES-rGO in coating.

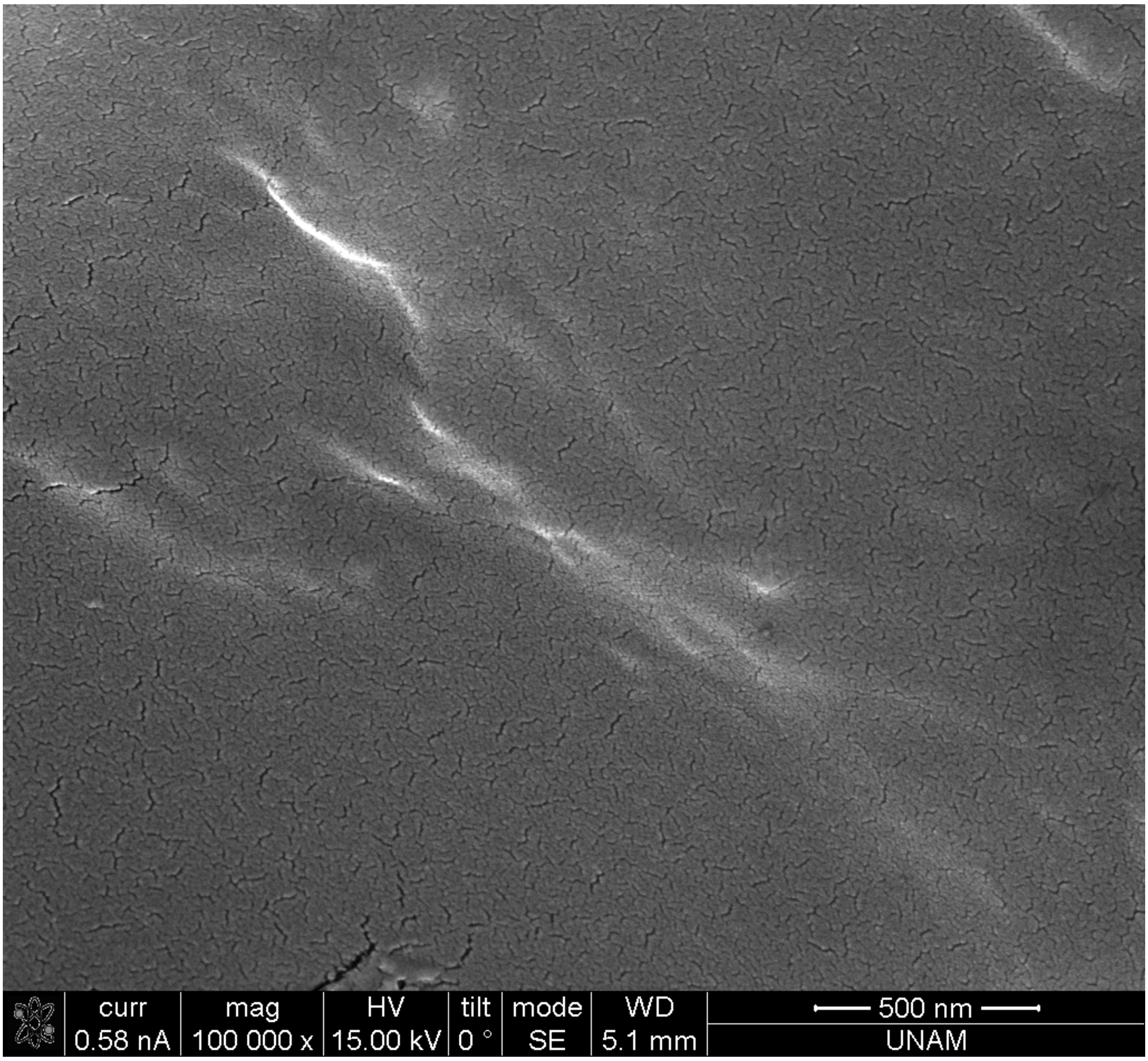

Figure S 27. SEM image of 10% APTES-rGO in coating.

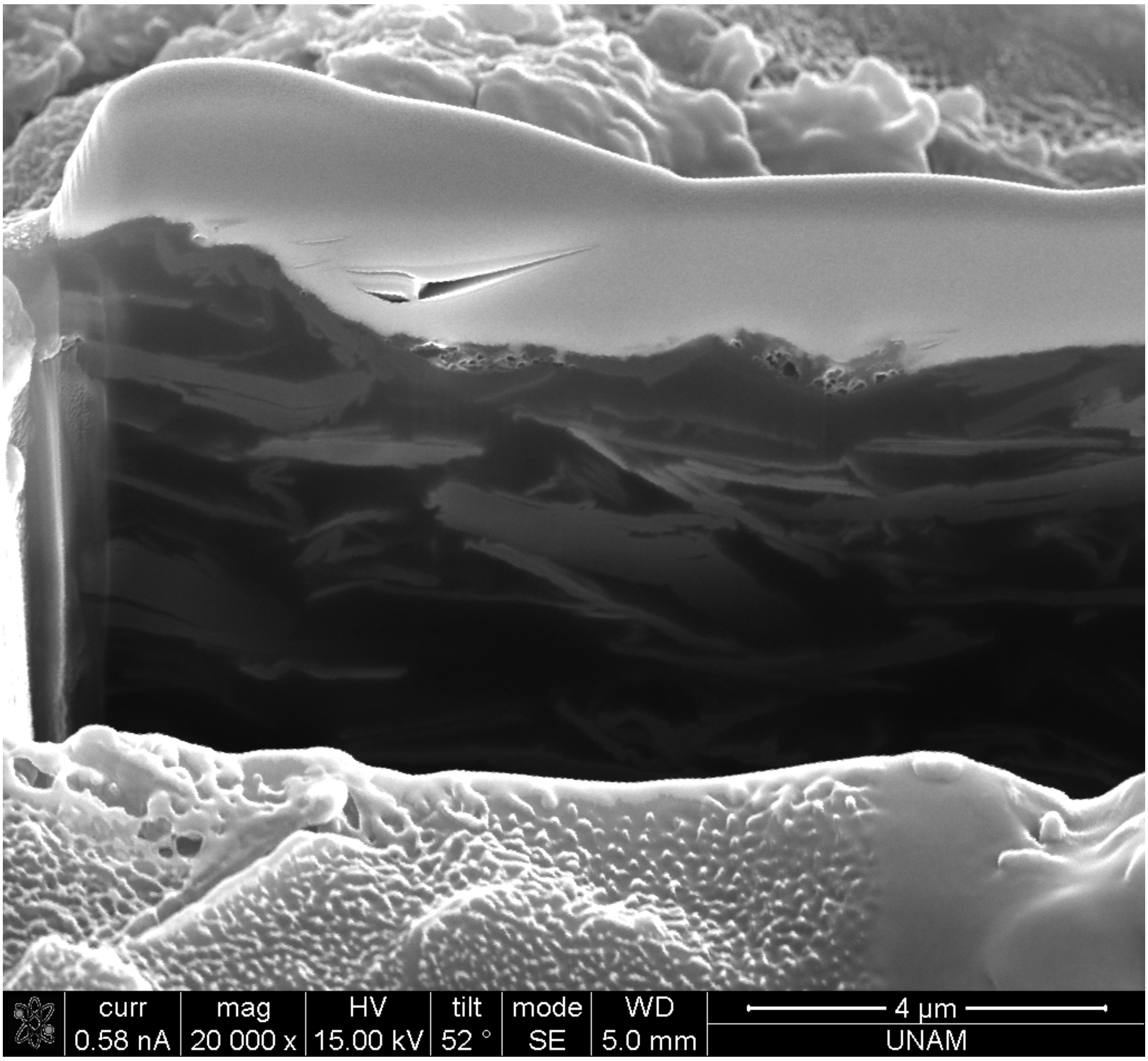

Figure S 28. SEM image of 10% APTES-rGO in coating.

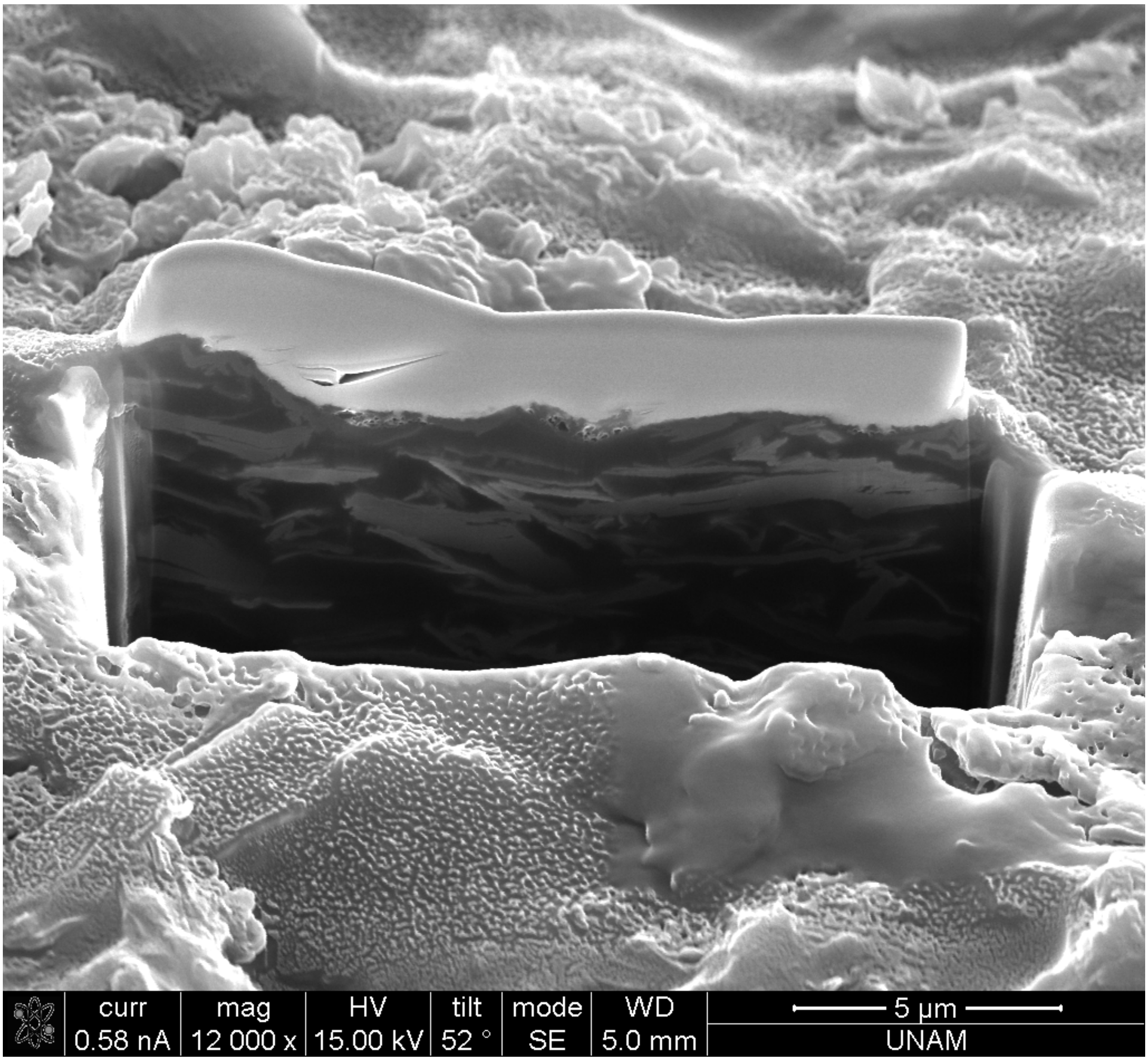

Figure S 29. SEM image of 10% APTES-rGO in coating.

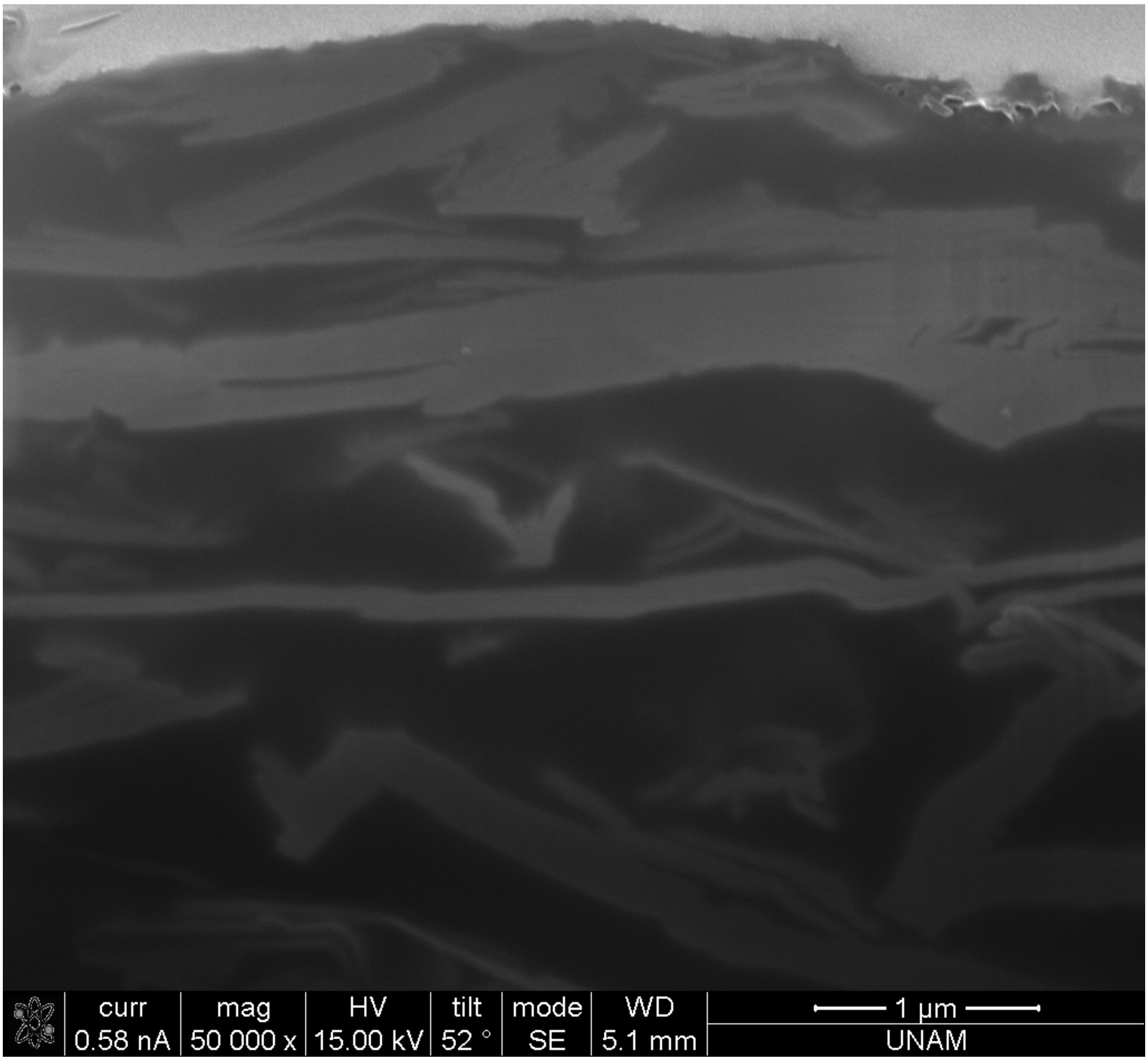

Figure S 30. SEM image of 10% APTES-rGO in coating.

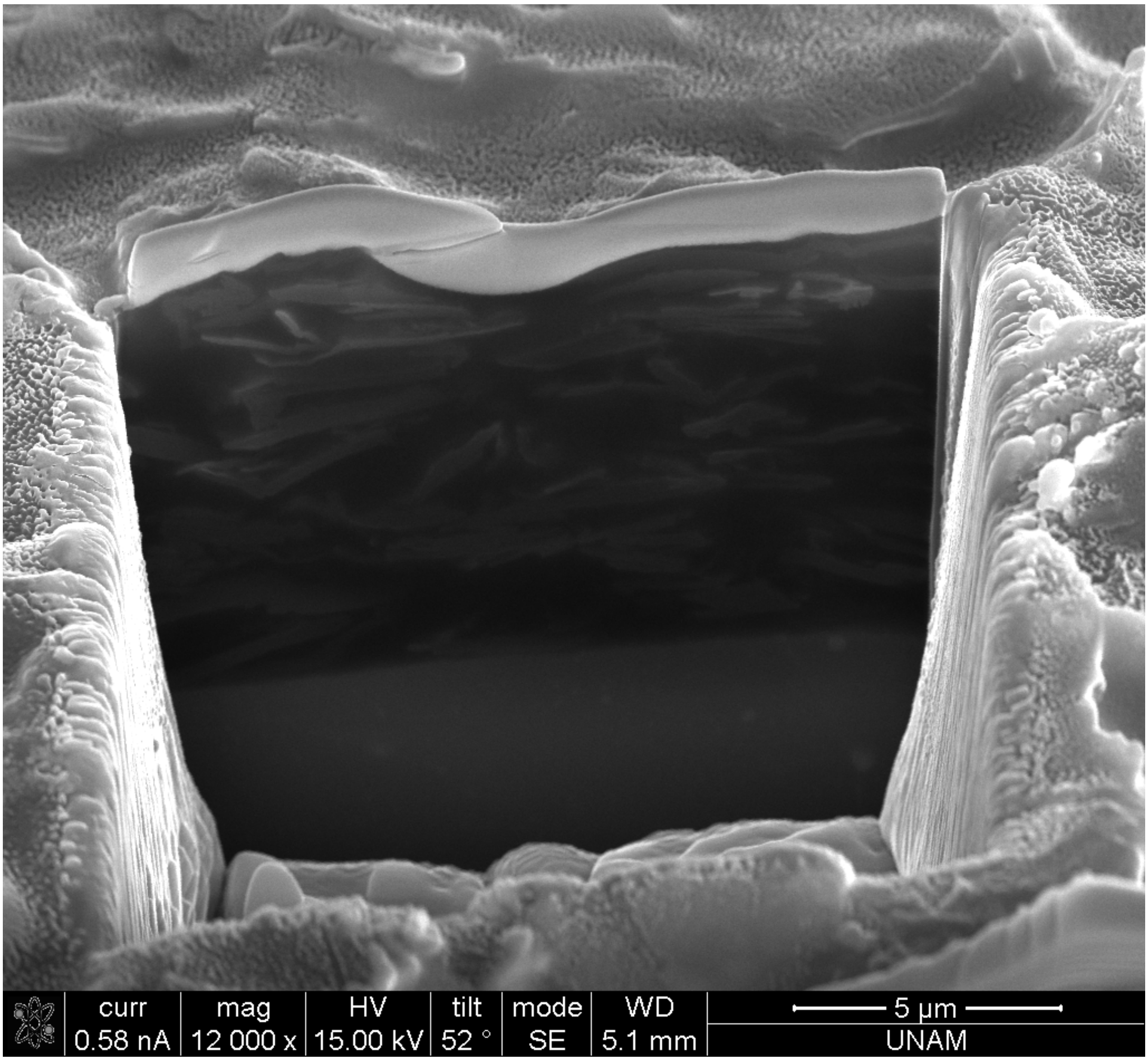

Figure S 31. SEM image of 10% APTES-rGO in coating.

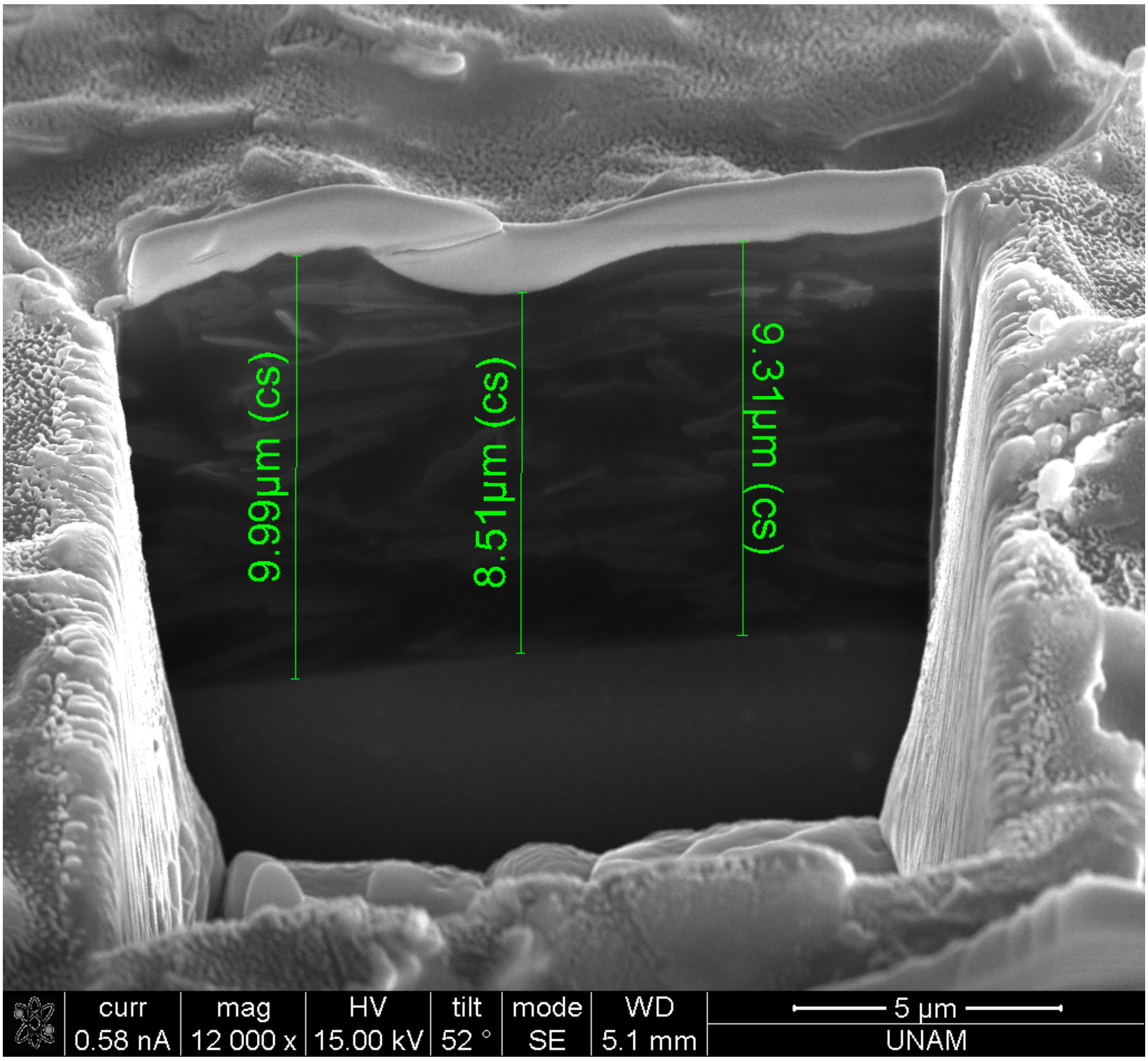

Figure S 32. SEM image of 10% APTES-rGO in coating.

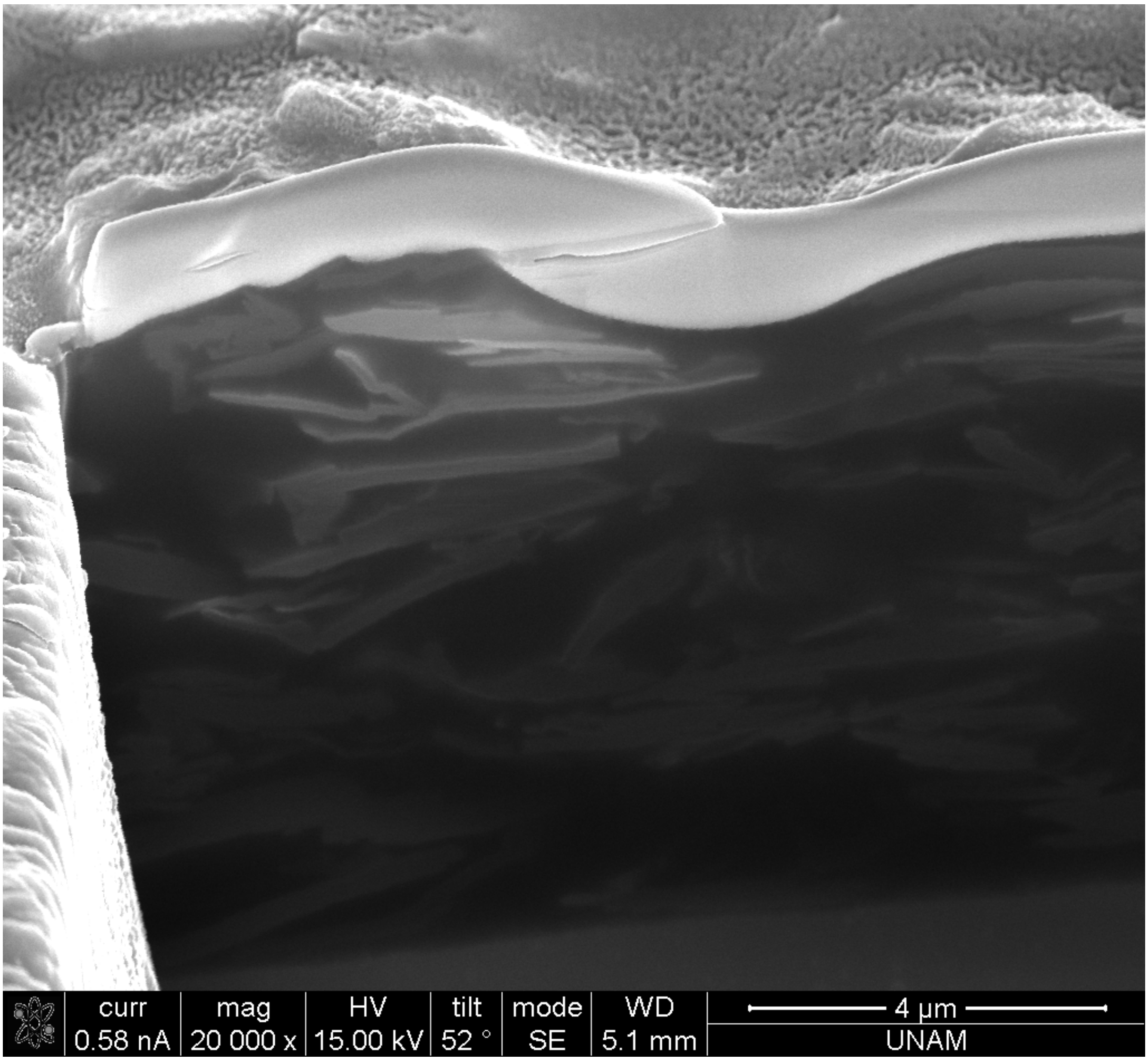

Figure S 33. SEM image of 10% APTES-rGO in coating.

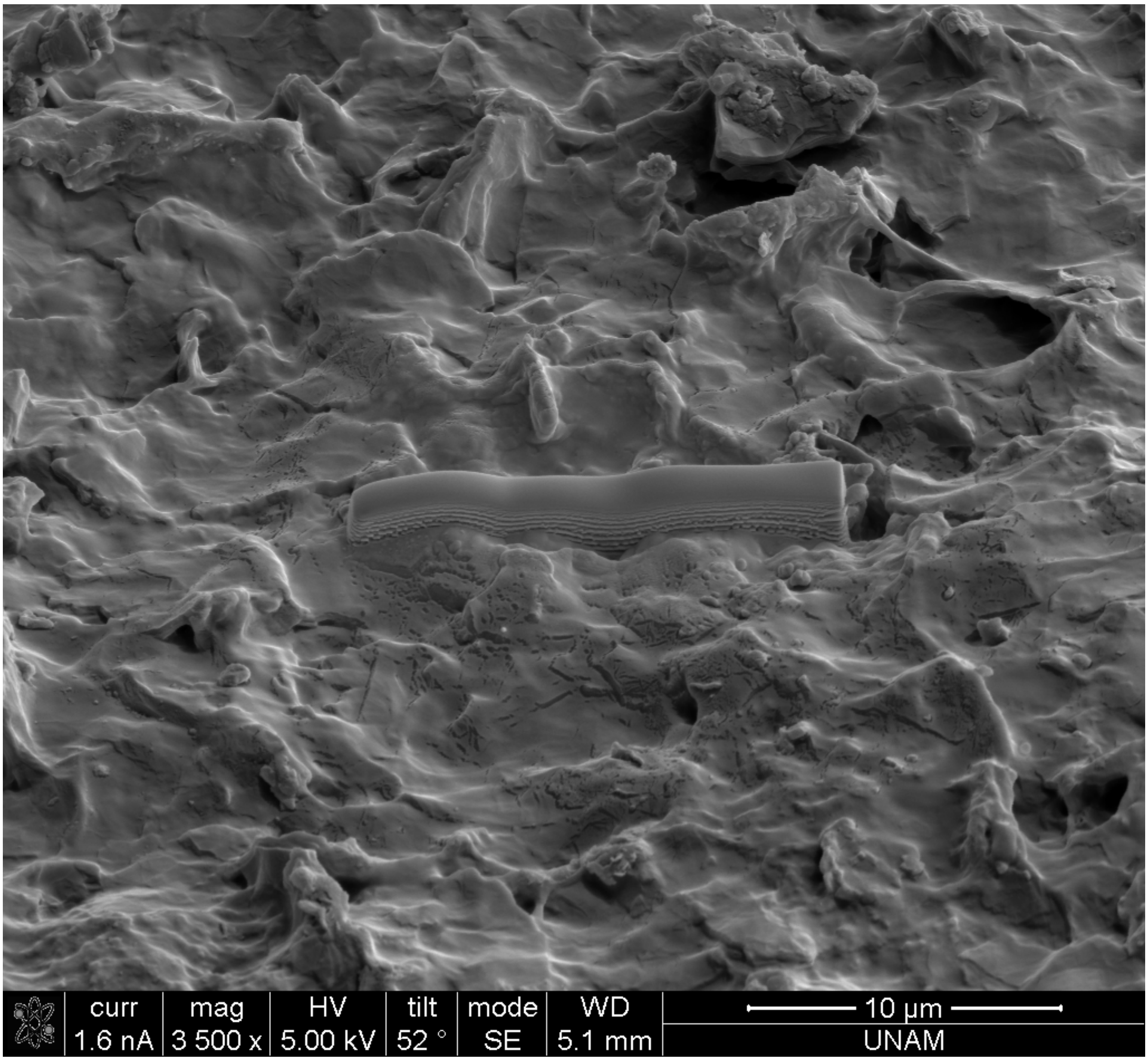

Figure S 34. SEM image of 5% rGO in coating.

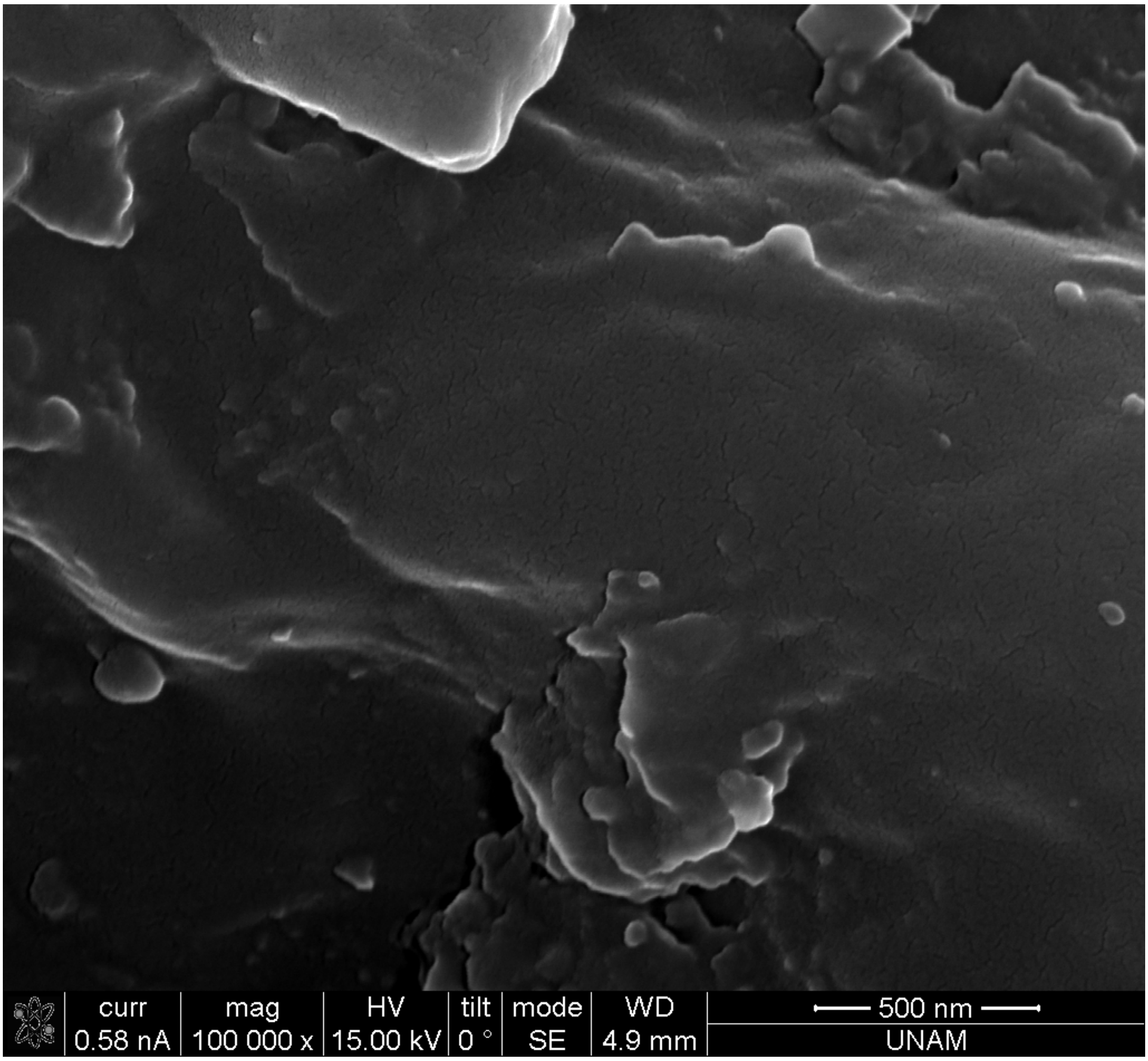

Figure S 35. SEM image of 5% rGO in coating.

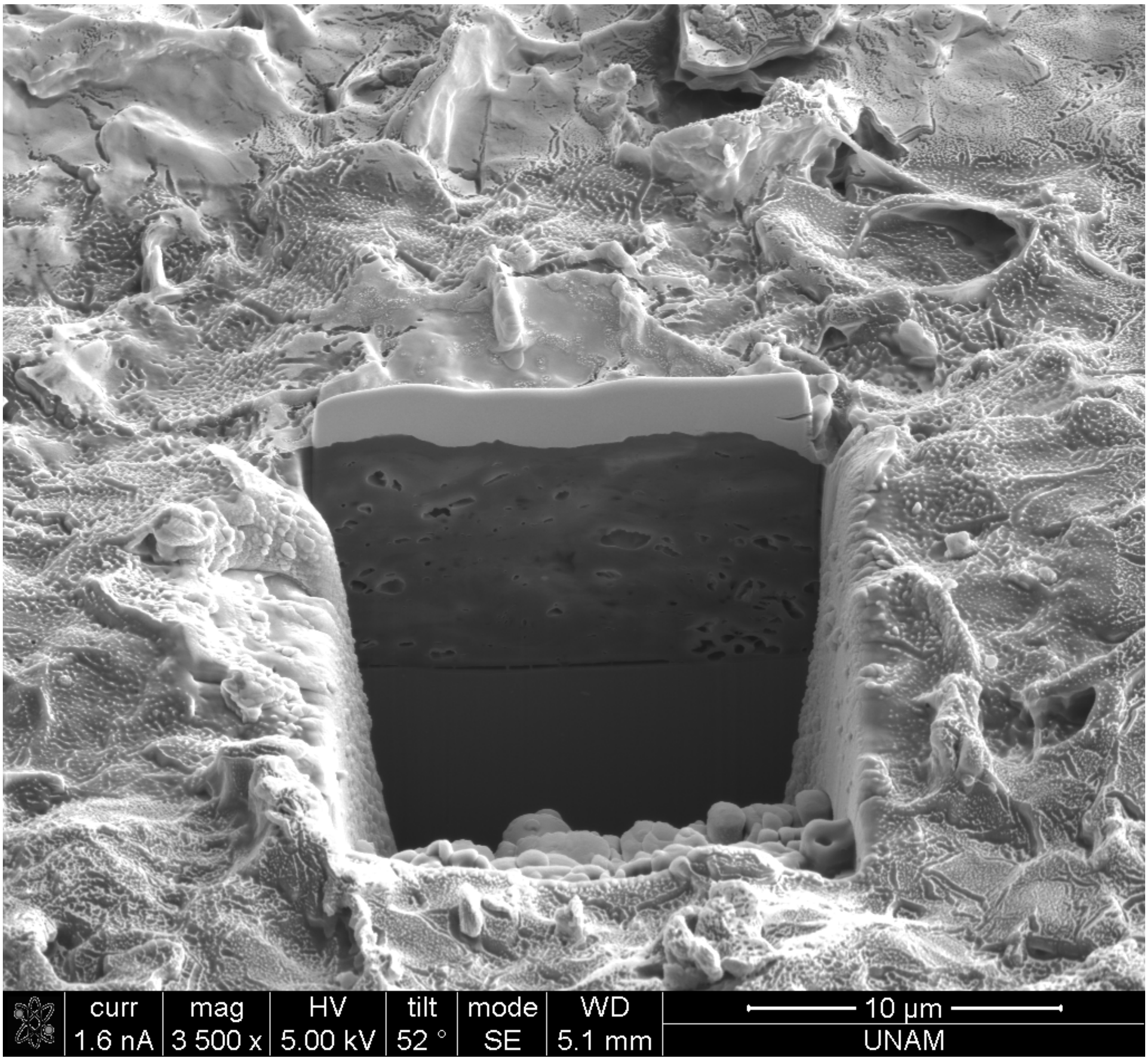

Figure S 36. SEM image of 5% rGO in coating.

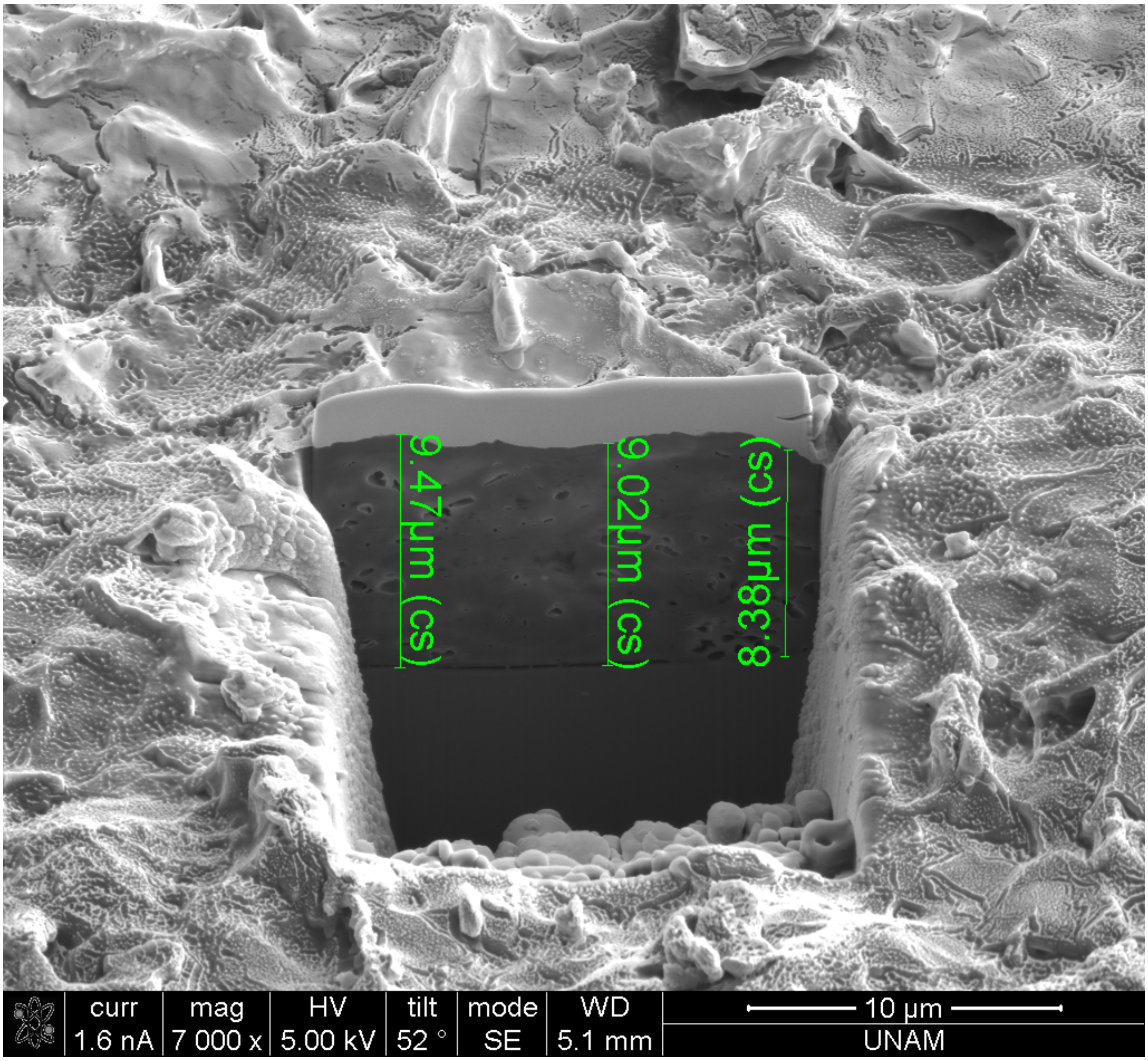

Figure S 37. SEM image of 5% rGO in coating.

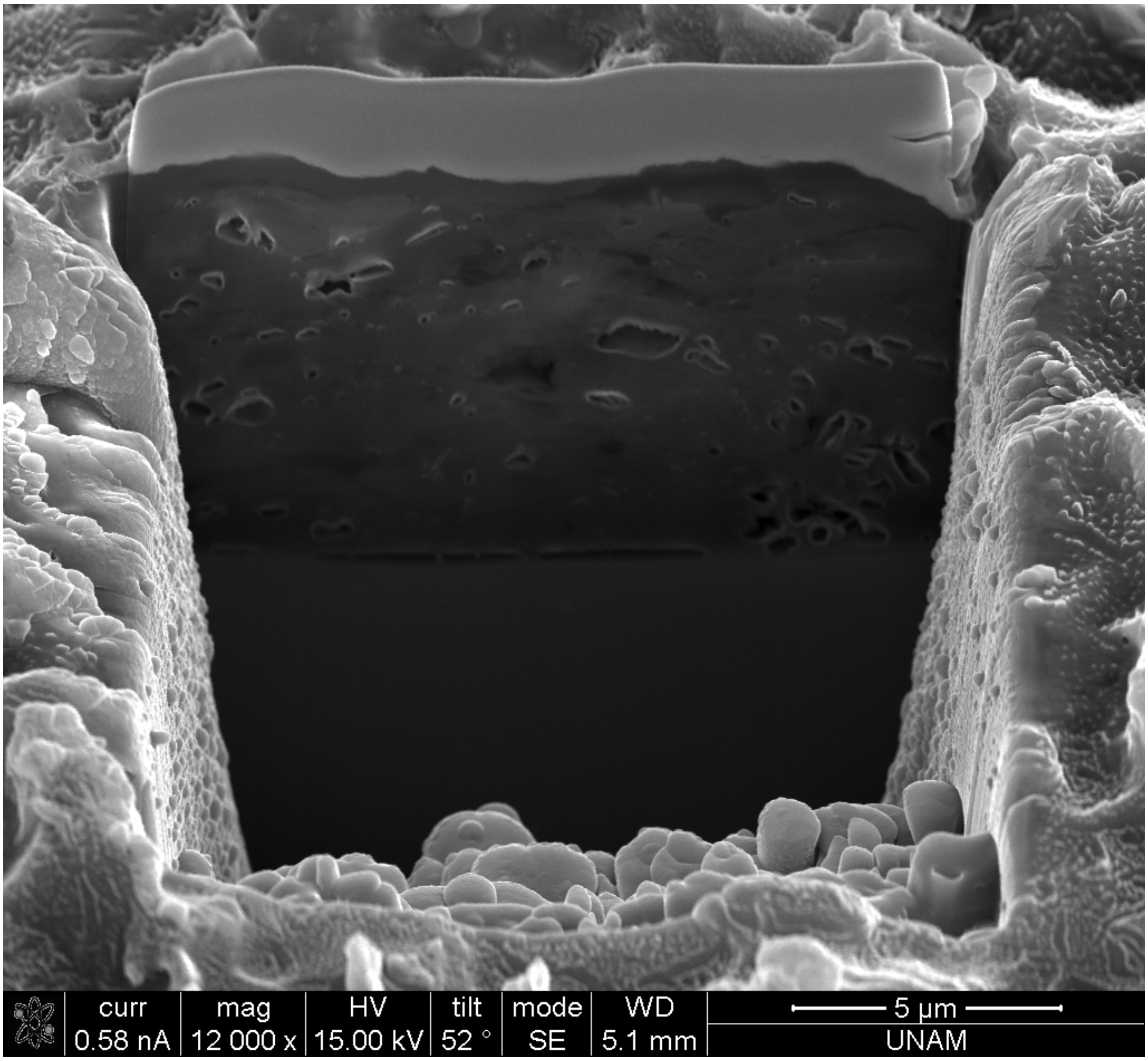

Figure S 38. SEM image of 5% rGO in coating.

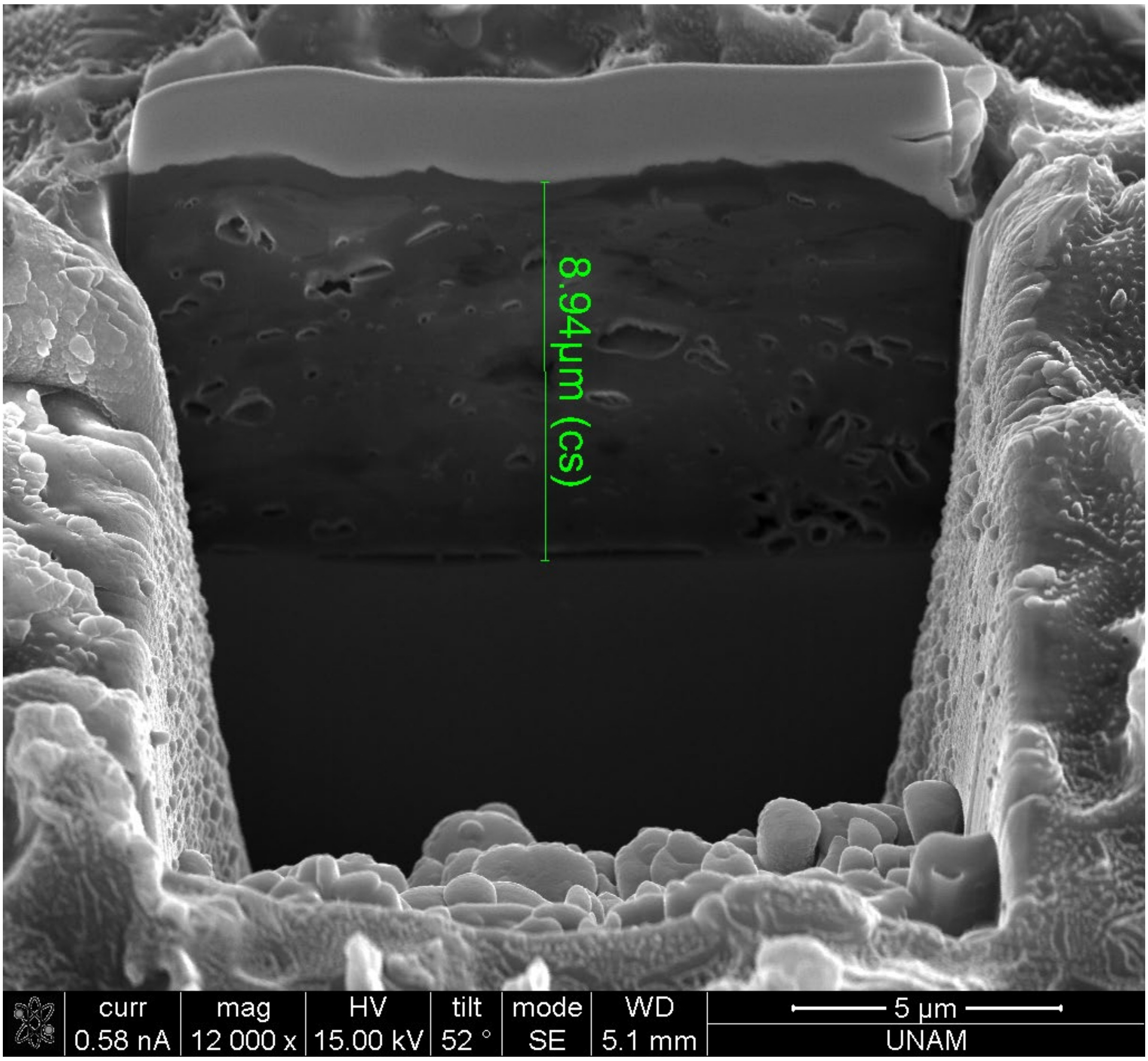

Figure S 39. SEM image of 5% rGO in coating.

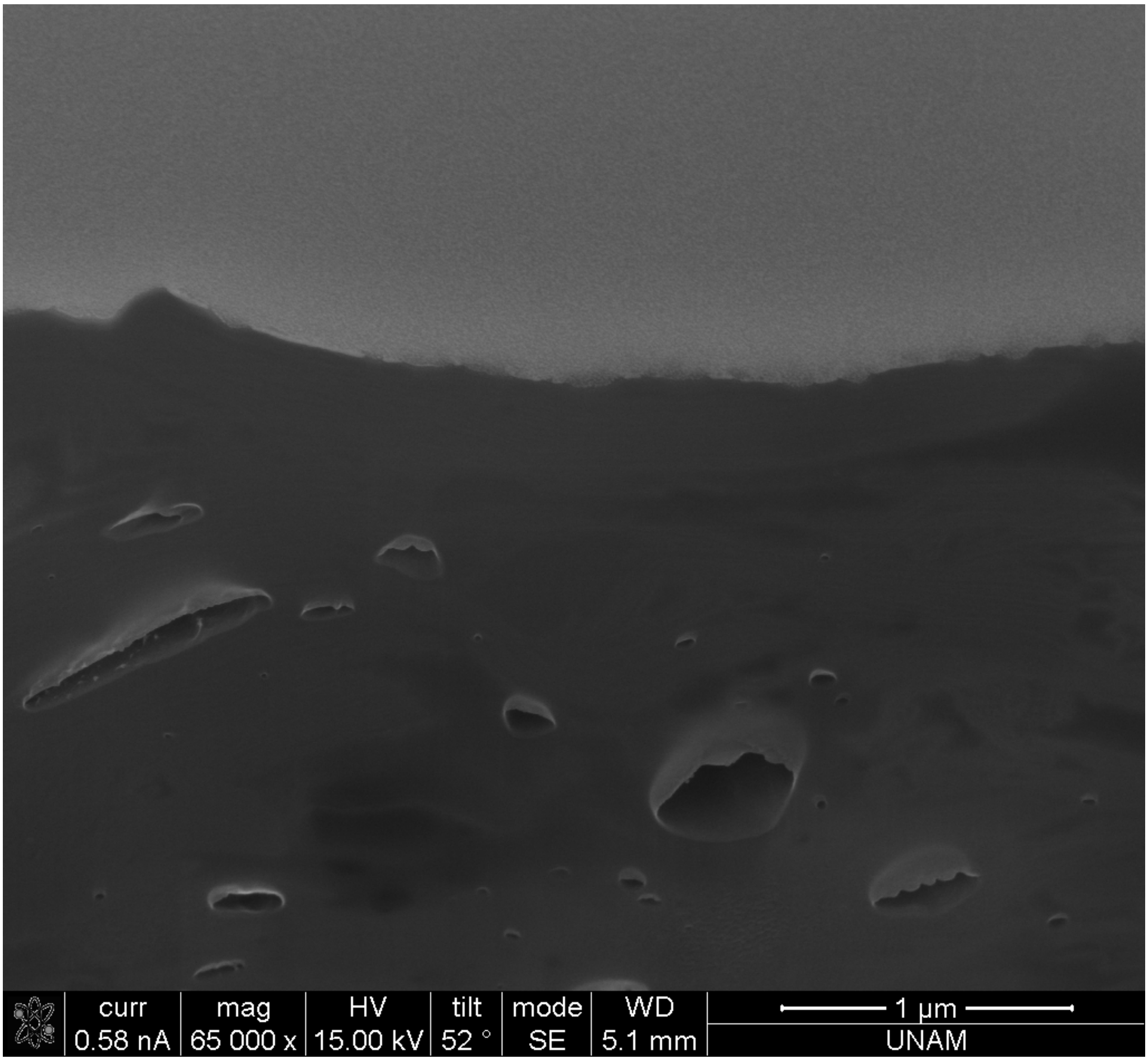

Figure S 40. SEM image of 5% rGO in coating.

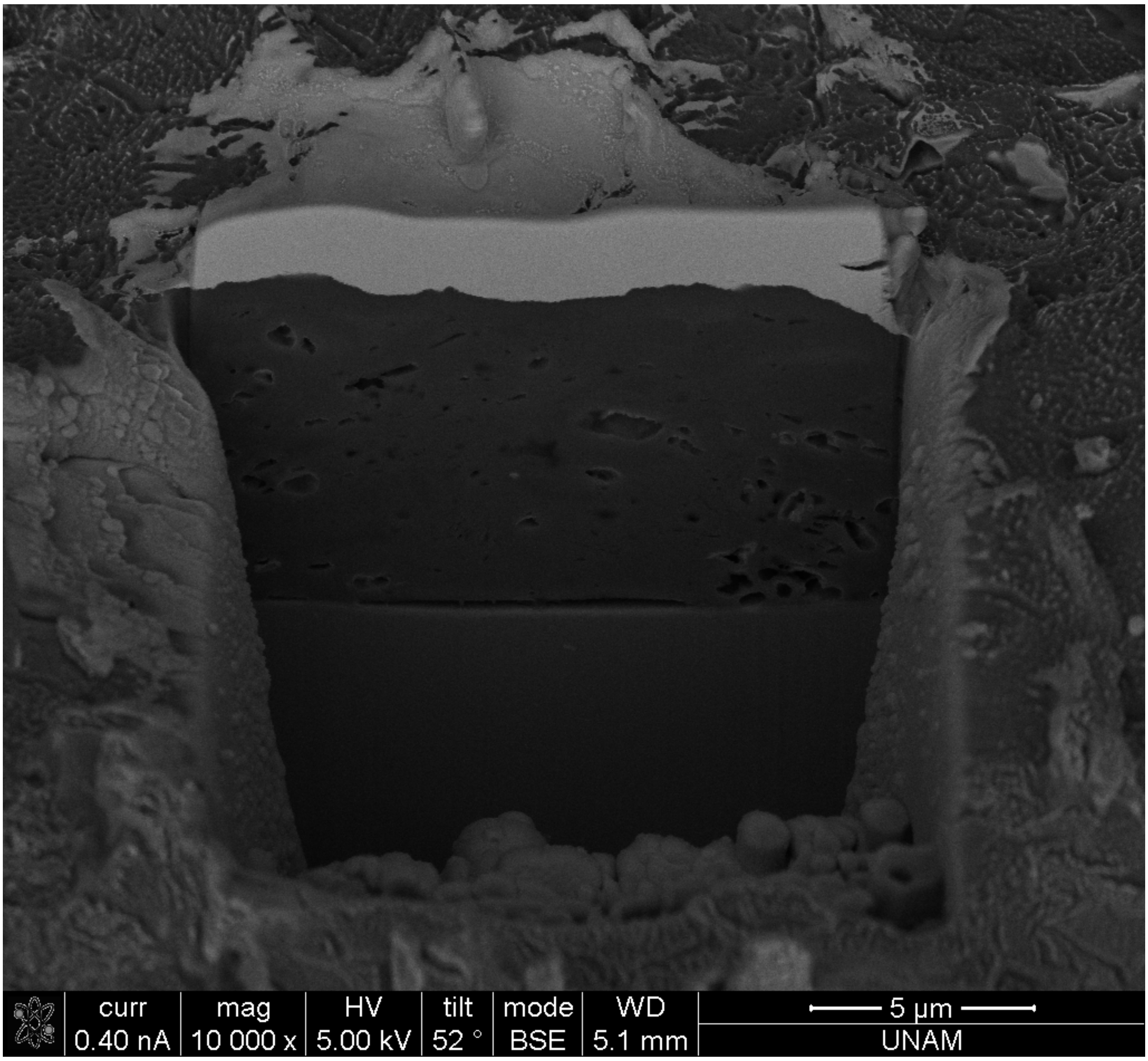

Figure S 41. SEM image of 5% rGO in coating.
